# A Systematic Review on the Viruses of *Anopheles* Mosquitoes: The Potential Importance for Public Health

**DOI:** 10.3390/tropicalmed8100459

**Published:** 2023-09-26

**Authors:** Juan C. Hernandez-Valencia, Paola Muñoz-Laiton, Giovan F. Gómez, Margarita M. Correa

**Affiliations:** 1 Grupo de Microbiología Molecular, Escuela de Microbiología, Universidad de Antioquia, Medellín 050010, Colombia; juan.hernandez21@udea.edu.co (J.C.H.-V.); paola.munoz1@udea.edu.co (P.M.-L.); gfgomezg@unal.edu.co (G.F.G.); 2 Dirección Académica, Escuela de Pregrados, Universidad Nacional de Colombia, Sede de La Paz, La Paz 202017, Colombia

**Keywords:** *Anopheles*, virus, virome, Insect-Specific Virus, arbovirus

## Abstract

*Anopheles* mosquitoes are the vectors of *Plasmodium*, the etiological agent of malaria. In addition, *Anopheles funestus* and *Anopheles gambiae* are the main vectors of the O’nyong-nyong virus. However, research on the viruses carried by *Anopheles* is scarce; thus, the possible transmission of viruses by *Anopheles* is still unexplored. This systematic review was carried out to identify studies that report viruses in natural populations of *Anopheles* or virus infection and transmission in laboratory-reared mosquitoes. The databases reviewed were EBSCO-Host, Google Scholar, Science Direct, Scopus and PubMed. After the identification and screening of candidate articles, a total of 203 original studies were included that reported on a variety of viruses detected in *Anopheles* natural populations. In total, 161 viruses in 54 species from 41 countries worldwide were registered. In laboratory studies, 28 viruses in 15 *Anopheles* species were evaluated for mosquito viral transmission capacity or viral infection. The viruses reported in *Anopheles* encompassed 25 viral families and included arboviruses, probable arboviruses and Insect-Specific Viruses (ISVs). Insights after performing this review include the need for (1) a better understanding of *Anopheles*-viral interactions, (2) characterizing the *Anopheles* virome—considering the public health importance of the viruses potentially transmitted by *Anopheles* and the significance of finding viruses with biological control activity—and (3) performing virological surveillance in natural populations of *Anopheles*, especially in the current context of environmental modifications that may potentiate the expansion of the *Anopheles* species distribution.

## 1. Introduction

Mosquitoes of the *Anopheles* genus are responsible for malaria transmission to humans [1], which, in 2020, caused the death of more than 600,000 people [2]. *Anopheles* mosquitoes also transmit the nematode *Wuchereria bancrofti*, the causing agent of filariasis in the tropics [3]; in addition, *Anopheles gambiae* and *Anopheles funestus* are the primary vectors of the O’nyong-nyong virus (ONNV), which causes fever and polyarthritis in Africa [4]. In general, species of this genus are not considered vectors of arboviruses; however, anthropophilic species that blood-feed on vertebrates are constantly exposed to circulating arboviruses; therefore, some *Anopheles* species may acquire and potentially spread viruses [5], mainly in regions of Latin America and Africa where fevers of unknown origin are common, and their etiological agents could be uncharacterized circulating arboviruses [6]. Despite the fact that *Anopheles* mosquitoes may potentially transmit arboviruses, their vector competence for viruses in general is uncertain; clarifying its vector role is a matter of public health importance [7,8].

Knowledge of the capability of *Anopheles* arbovirus transmission is relevant in the current context of overpopulated human settlements, where anthropogenic activities crossover from human settings into the natural environment [9,10], which may promote human–mosquito interaction propitiating virus transmission [11]. Although *Anopheles* has not been formally incriminated in the transmission of arboviruses other than ONNV, some studies suggest that various species may transmit arboviruses such as the Rift Valley fever virus (RVFV) in Africa [12], the Mayaro virus (MAYV) in Central and South America [13] and the Japanese encephalitis virus (JEV) in the Asiatic southeast [14].

Advancement in massive sequencing technologies and the emergence of metagenomics has allowed the characterization of the virome of various organisms, including some mosquito species [15]. As a result, the knowledge of the viral communities circulating in mosquito populations has greatly increased in recent years. The evidence indicates that most of the viruses are Insect-Specific Viruses (ISVs). Specifically, in *Anopheles* mosquitoes, some ISVs showed a close phylogenetic relationship with medically relevant arboviruses, which suggested the probable emergence of arboviruses from ISVs [7]. In addition, the evolutionary plasticity of RNA viruses indicates that they may originate new arboviruses, which has public health implications [16,17]. The study of vertically transmitted ISVs, which cause prolonged infections in mosquito populations, has gained attention as a potential tool for viral paratransgenesis and biological control [16].

The study of the viruses harbored and potentially transmitted by *Anopheles* is a relevant matter with implications in public health, either in the case of transmission of pathogenic viruses to humans or for the potential utility of appropriate viruses as biological control agents. Therefore, this systematic literature review was carried out to identify research studies that detected viruses in natural *Anopheles* populations or evaluated infection or transmission capacity in laboratory-reared mosquitoes.

## 2. Materials and Methods

A systematic literature review was performed following recommendations by the Preferred Reporting Items for Systematic Reviews and Meta-Analyses (PRISMA) guide [18].

### Scientific Literature Selection and Data Extraction

Identification: The scientific literature on the topic was reviewed from 1935 (the date of the first published study related to viruses in *Anopheles* mosquitoes) to November 2021 using five databases: EBSCO-Host, Google Scholar, Science Direct, Scopus and PubMed. The search terms were (i) EBSCO-Host: TI = *Anopheles* AND TI = virus OR AB *Anopheles* AND AB virus; (ii) Google Scholar: allintitle: *Anopheles* virus, allintitle: *Anopheles* virome; (iii) Science Direct: (Find articles with these terms: *Anopheles*)/(Title, abstract or author-specified keywords: virus), (Find articles with these terms: *Anopheles*)/(Title, abstract or author-specified keywords: virome); (iv) Scopus: (TITLE-ABS-KEY (*Anopheles*) AND TITLE-ABS-KEY (virus) OR TITLE-ABS-KEY (virome)) AND (LIMIT-TO (DOCTYPE, “ar”) OR LIMIT-TO (DOCTYPE, “sh”)); y (v) Pubmed: (*Anopheles* [Title/Abstract]) AND (virome [Title/Abstract]), (*Anopheles* [Title/Abstract]) AND (virus [Title/Abstract]). The articles obtained were imported to the Rayyan QCRI web server (https://www.rayyan.ai/ (accessed on 25 August 2023)) [19], and duplicates were manually removed.

Scientific literature screening: Documents not fulfilling the following criteria were excluded: an original article addressing the study of viruses in *Anopheles* mosquitoes and availability of the full article. To ensure reproducibility, two researchers conducted the article search, selection and screening independently; after comparing their results, they resolved disagreements by consensus.

Data extraction: Data extraction was performed on articles that met the inclusion criteria, i.e., virus detection in *Anopheles* natural populations and infection or transmission in laboratory-reared mosquitoes. Articles related to *Anopheles* cell lines were excluded. The following variables were compiled from each article: main author, publication date, study type (field, semi-field or laboratory), study location, geographical coordinates for field studies, collection date, *Anopheles* species studied, mosquito sex, number of mosquitoes analyzed, number of mosquitoes per pool, number of pools positive for viruses, viral detection method, viral species and taxonomic assignation, and viral group classification (arbovirus, probable arbovirus, ISV or other viruses known to infect vertebrates, plants and prokaryotic organisms). The location of the mosquito collection site was used for studies that did not report geographic coordinates. The taxonomy of the viruses was defined according to taxonomic rules of the International Committee on Taxonomy of Viruses (ICTV) (https://ictv.global/taxonomy (accessed on 25 August 2023)). Arbovirus and probable arbovirus status were specified according to the International Catalog of Arboviruses (Arbocat) (https://wwwn.cdc.gov/arbocat/ (accessed on 25 August 2023)), which is based on the criteria of the Subcommittee on the Evaluation of Arthropod-Borne Status [20].

The viruses found in wild-caught *Anopheles* worldwide were georeferenced using ArcGIS 10.8.2. Other figures were generated using Microsoft Excel and Past 4.11.

## 3. Results

### 3.1. Search Results

**Figure 1:**
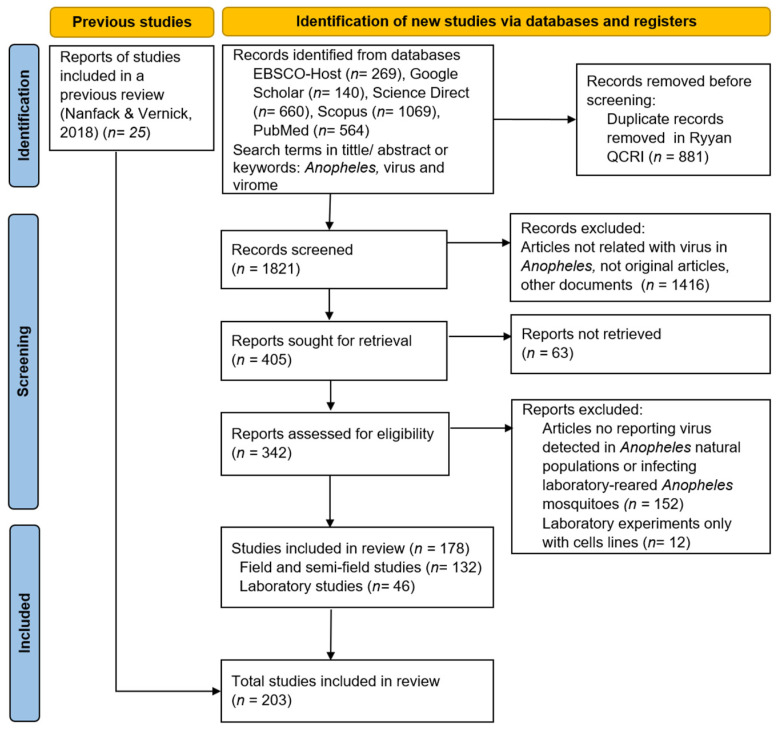
PRISMA flow diagram of search and selection of studies related to viruses in *Anopheles* mosquitoes [21].

A total of 2702 articles were retrieved from the databases; after exclusion by screening, 342 were considered, and from these, 164 were discarded for not fulfilling the inclusion criteria. Finally, 203 articles related to viruses detected in *Anopheles* natural populations or infecting laboratory-reared *Anopheles* mosquitoes were included. In addition, 25 articles from a previous systematic review were added, along with prior data revision of the reports and criteria fulfillment [21] (Figure 1).

### 3.2. Viruses Detected in Anopheles Mosquitoes

According to the data analyzed from the first report dating from 1935 until November 2021, 161 viruses in 54 *Anopheles* species from 41 countries were identified. Furthermore, viral infection or transmission in laboratory-reared mosquitoes was demonstrated for 28 viruses in 15 *Anopheles* species (Table S3). Worldwide, most of the studies on *Anopheles* viral infection have been conducted in Asia-Oceania (44.2%) and the American continent (26.2%), where most are from the USA, followed by Africa (22.8%). Regarding the mosquito sex, 79% of the studies were conducted in *Anopheles* females, 3.3% in both sexes and 0.28% in males; 16% of the studies did not report the mosquito sex.

**Figure 2:**
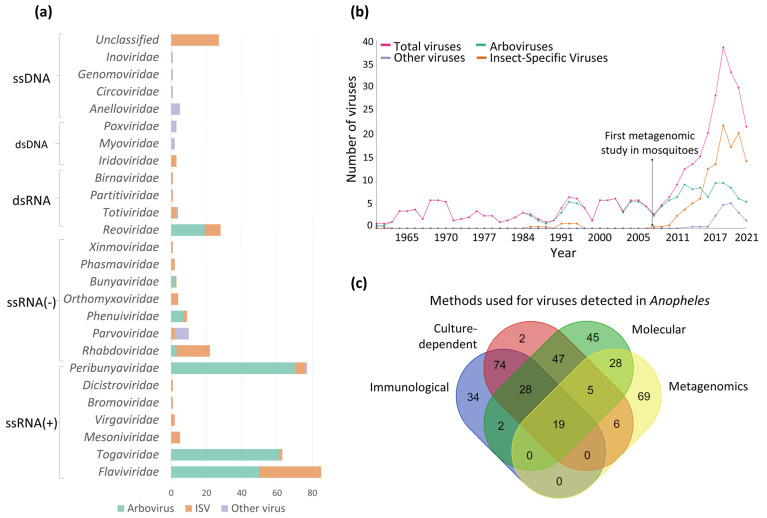
Overview of viruses detected in *Anopheles* mosquitoes worldwide. (**a**) Number of detections of arboviruses, Insect-Specific Viruses (ISVs) and other viruses (viruses of vertebrates, plants and prokaryotes), grouped by viral family; (**b**) timeline of the number of viruses detected in *Anopheles*; (**c**) the Venn diagram shows the number of viruses detected in the *Anopheles* per detection method or in combination.

The viruses detected in *Anopheles* mosquitoes belong to various DNA and RNA viral families, the latter being the most prevalent in natural populations of *Anopheles* (Figure 2). The most frequently reported viral families were *Flaviviridae*, *Peribunyaviridae*, *Togaviridae* and *Reoviridae* (Figure 2a). Of the 161 viruses detected in wild *Anopheles*, 35 were arboviruses, 24 were probable arboviruses, 84 were ISVs, 12 were viruses that infect vertebrates, 4 infected plants and 2 infected prokaryotic organisms.

Viral detection in *Anopheles* was carried out by methods such as culture-dependent, immunological, molecular and metagenomics. For decades, the combination of culture-dependent and immunological methods allowed the detection of a high number of viruses in *Anopheles*; however, in just 14 years of the 354 viral reports, 127 were achieved with omics technologies, and most of them correspond to ISVs. The former demonstrates a trend in the discovery of ISVs after the appearance of massive sequencing technologies (Figure 2b,c).

#### 3.2.1. Arboviruses and Probable Arboviruses Detected in *Anopheles*

**Figure 3:**
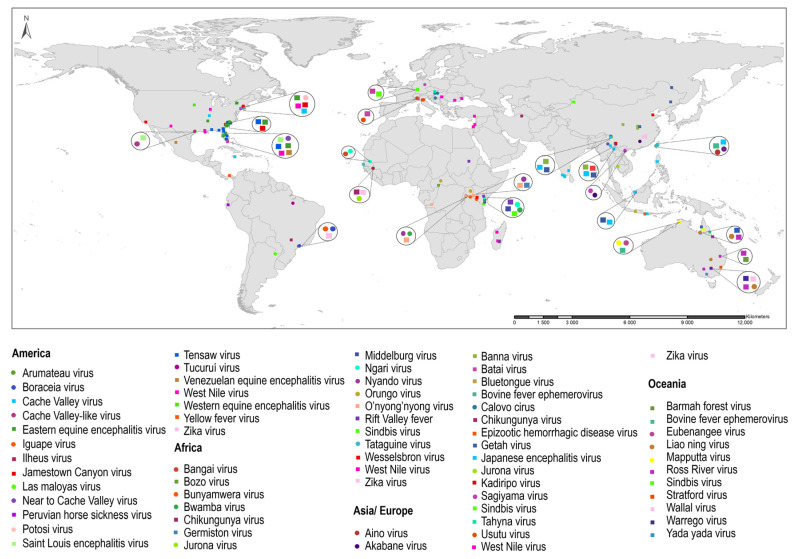
Worldwide distribution of arboviruses (square) and probable arboviruses (circles) detected in wild-caught *Anopheles*.

Thirty-five arboviruses and twenty-four probable arboviruses were reported in *Anopheles* (Table 1 and Table S1). The arboviruses more frequently detected were the Eastern equine encephalitis virus (EEEV), Tensaw virus (TENV), West Nile virus (WNV), Japanese encephalitis virus (JEV), Ross River virus (RRV) and the O’nyong-nyong virus (ONNV). The arboviruses families more often detected are *Peribunyaviridae*, *Togaviridae*, *Flaviviridae* and *Reoviridae* (Figure 2a). The studies reporting the highest number of arboviruses and probable arboviruses in wild-caught *Anopheles* were conducted with mosquitoes collected in the USA (30.5%), Australia (12.4%), China (10.0%) and Kenya (7.1%) (Figure 3, Table S1).

##### The *Peribunyaviridae* Family

Most of the arboviruses detected in natural populations of *Anopheles* mosquitoes correspond to the *Peribunyaviridae* family, with 23 reports, all belonging to the *Orthobunyavirus* genus. The most frequently reported viruses were TENV, Batai (BATV) and Cache Valley virus (CVV). TENV was detected 18 times among *Anopheles crucians* and *Anopheles quadrimaculatus* in the states of Florida, Georgia and South Carolina in the USA; detections were performed during surveillance campaigns of arboviruses in mosquitoes [31,33] (Table 1). Also, TENV was evaluated in laboratory-reared *Anopheles quadrimaculatus* and *Anopheles albimanus*; these mosquitoes showed susceptibility to virus infection and transmission [141] (Table 2). CVV, an arbovirus distributed in Central and North America, was reported seven times, mainly in *An. quadrimaculatus* and *Anopheles punctipennis* from the USA. In addition, infection susceptibility and transmission capacity of this virus was demonstrated in *An. quadrimaculatus* and *An. punctipennis* [142,143] (Table 2). Finally, BATV was reported six times in Italy and Germany, mainly in *Anopheles maculipennis* (Table 1).

Less frequently detected orthobunyaviruses include Bwamba virus (BWAV) and Bunyamwera virus (BUNV), both of which are endemic arboviruses in East Africa. They were detected in *An. gambiae*, *An. funestus* and *Anopheles coustani* from Kenya [113,115,124]; also, a single detection of BWAV was reported in *An. funestus* from Uganda [66]. Laboratory-reared *An. gambiae* showed infection susceptibility for both viruses and transmission capacity for BUNV [144,145] (Table 2). In addition, Jamestown Canyon virus (JCV), Germiston virus (GERV), Bozo virus (BOZOV) and Tahyna virus (TAHV) were detected in *Anopheles*; although, there were no studies evaluating infection or transmission in laboratory-reared mosquitoes.

**Table 2:** Viruses that may potentially be transmitted by *Anopheles* mosquitoes according to the vector incrimination criteria **^+^**.

Virus Name (Abbreviation)	*Anopheles* Species	Detected in Natural Populations (Country/Number of Detections)	Detected during an Outbreak (Yes/No)	Results of Laboratory Studies (Viral Infection and Transmission)	References *
O’nyong-nyong virus ★ (ONNV)	*An. gambie*	Uganda/2, Kenya/1	Yes [65]	IR 75% at 7 dpi with recombinant virus, TR not determined	[4]
				Infection, IR not available, TR not determined	[146]
				Limited infection and spread, with no differences between transgenic and wild mosquitoes, TR 0%	[147]
				Studies with a recombinant virus, IR 78%, DR 15% at 6 dpi; IR 84%, DR 25% at 8 dpi, TR not determined	[148]
				IR 75%, TR 0% at 7 dpi; IR 95%, TR 57% at 14 dpi	[149]
Rift Valley fever virus (RVFV)	*An. coustani*	Madagascar/1, Sudan/1	Yes [12,74]	IR 50%, TR 100% at 8 dpi	[150]
Saint Louis encephalitis virus (SLEV)	*An. quadrimaculatus*	USA/1	Yes [106]	Infection (IR not determined), transmission 0%	[151]
Tensaw virus (TENV)	*An. quadrimaculatus*	USA/4	No	IR 100% at 10 and 20 dpi, transmission 20% at 14 dpi	[141]
Japanese encephalitis virus (JEV)	*An. subpictus*	India/4 ×	Yes [54,55]	N/A	N/A
West Nile virus (WNV)	*An. punctipennis*	USA/3	Yes [46,47]	N/A	N/A
	*An. maculipennis*	Romania/1, Serbia/1	Yes [37,40]	N/A	N/A
Bunyamwera virus (BUNV)	*An. gambiae*	Kenya/1	No	IR 38%, transmission 71% at 14 dpi	[144]
Cache Valley virus (CVV)	*An. quadrimaculatus*	USA/3	No	IR 100%, transmission 20% at 7 dpi; IR 100%, transmission 33% at 14 dpi	[142]
			No	IR 100%, TR 0% at 10–19 dpi	[143]
	*An. punctipennis*	USA/2	No	IR 85%, TR 30% at 14–18 dpi	[143]
Eastern equine encephalitis virus (EEEV)	*An. quadrimaculatus*	USA/5	No	Infection rate not determined; transmission 40% at 10 dpi, 50% at 11 dpi	[152]
Myxoma virus § (MYXV)	*An. atroparvus*	England/1	Yes [153]	Infectious virion up to 220 dpi in mosquito mouthparts	[154]

Abbreviations: N/A, no laboratory studies were found; IR, infection rate is the percentage of engorged females with viral particles in the body; DR, dissemination rate is the percentage of engorged females with viral particles in legs/wings; TR, transmission rate is calculated as percentage of engorged females with viral particles in the saliva/salivary glands; dpi, days post-infection. ★ The *Anopheles* mosquito is the confirmed vector. × Detected in males and females during virus outbreaks. § Myxoma virus is not an arbovirus, but there is evidence of its mechanical transmission by *Anopheles* to rabbits. + Vector incrimination criteria: 1. Virus recovery from mosquito natural populations, 2. Evidence of mosquito contact with the vertebrate host, 3. Virus outbreaks and vector co-occurrence in space and time, and 4. Proof of virus transmission under laboratory conditions [155]. * Table S3 displays additional studies that evaluated virus infection and transmission in laboratory-reared *Anopheles* [156,157,158,159,160,161,162,163,164,165,166,167,168,169,170,171,172,173,174,175,176,177,178,179,180,181,182,183,184].

##### The *Togaviridae* Family

*Anopheles* is a recognized primary vector of the O’nyong-nyong virus (ONNV) of the *Togaviridae* family. This virus has been detected in *An. gambiae* and *An. funestus* in Africa (Table 1 and Figure 3). In addition, the capacity of laboratory-reared *An. gambiae* to maintain the ONNV infection was demonstrated, and one study reported ONNV dissemination to the mosquito salivary glands (Table 2). Moreover, the Sindbis virus (SINV) was detected in different *Anopheles* species from Australia, Kenya, China and Germany (Table 1). Infection with SINV was reported in laboratory-reared *Anopheles freeborni* [181] and in *An. albimanus*, which also showed virus transmission capacity [180] (Table S3).

The viruses that cause equine and human encephalomyelitis, Eastern equine encephalitis virus (EEEV), Venezuelan equine encephalitis virus (VEEV) and Western equine encephalitis virus (WEEV), have also been detected in *Anopheles* natural populations (Table 1 and Figure 3). EEEV was reported 20 times among *An. crucians*, *An. quadrimaculatus* and *An. punctipennis* during arbovirus surveillance campaigns or virus outbreaks in the USA (Table S3). Laboratory studies demonstrated infection of *An. punctipennis* with EEEV [165], and *An. albimanus* and *An. quadrimaculatus* were competent for transmission [152,165]. Regarding VEEV, it has been detected in *An. crucians* in the USA and *Anopheles pseudopunctipennis* in Mexico. A laboratory study demonstrated *An. albimanus* infection susceptibility and transmission competence for this virus [184]. Finally, WEEV was detected in *An. punctipennis* during an arbovirus surveillance campaign in Iowa, USA [136] (Table S3).

Chikungunya virus (CHIKV) was reported in arbovirus surveillance studies in *An. gambiae* from Senegal and *An. maculipennis* from Iran [122,123]. Also, infection susceptibility to CHIKV was reported in laboratory-reared *An. albimanus* [162], and infection susceptibility and transmission capacity in *Anopheles stephensi* [163] (Table S3).

Other alphaviruses detected in *Anopheles* natural populations are the Getah virus (GETV), Ross River virus (RRV), Barmah Forest virus (BFV), Middelburg virus (MDIV) and Yada yada virus (YYV). RRV is endemic in Australia and other South Pacific islands; there are nine detection reports among *Anopheles amictus*, *Anopheles annulipes* and *Anopheles bancroftii*, all during surveillance campaigns of arboviruses in mosquito populations in Australia. Moreover, GETV, a horse and pig pathogen, was detected on seven occasions among *Anopheles hyrcanus*, *Anopheles sinensis* and *Anopheles* spp.; the first report of GETV was from 1974 in Russia and Malaysia [79,80], and the other six in *An. sinensis* from China between 2009 and 2021. Finally, BFV, MDIV and YYV were detected only once in *Anopheles*; MDIV was detected in *An. coustani* from Kenya and BFV and YYV in *An. annulipes* and *An. amictus,* respectively, both in Australia (Table S1 and Figure 3).

##### The *Flaviviridae* Family

The West Nile virus (WNV) of the *Flaviviridae* family was detected 17 times in 12 *Anopheles* species in various countries of Africa, America, Asia and Europe (Table 1 and Figure 3); 8 of these were from the USA during WNV outbreaks occurred between 2000 and 2002 in the states of New York and Illinois; although, the detections were conducted later, between 2004 and 2010, 3 of them in *An. punctipennis* [46,47]. Three detections of WNV in *An. maculipennis* and *An. hyrcanus* were during outbreaks in Serbia and Romania [39,40]. During this systematic review, no laboratory studies were found that evaluated WNV infection and transmission in *Anopheles* species.

Other flaviviruses of the same WNV serocomplex, such as Japanese encephalitis virus (JEV), Saint Louis encephalitis virus (SLEV) and Usutu virus (USUV), have also been detected in *Anopheles* natural populations (Table 1 and Figure 3). JEV, the most important etiologic agent of human encephalitis, was identified 16 times in eight *Anopheles* species in Asia; four of these in *Anopheles subpictus* and two during virus outbreaks in Alappuzha and Cuddalore districts in India; and of note, JEV was also recovered from *An. subpictus* males [54,55] (Table 2). There are four JEV reports on *An. sinensis* from China; the first was in 1987 [48], and the most recent one was in 2018 [49]. In addition, there were five JEV detections in various *Anopheles* species from Southeast Asia [52,56,57,58]. Regarding SLEV, it was reported three times in *An. crucians* and *An. quadrimaculatus* in encephalitis outbreaks that occurred during the 1960s in Florida and Texas in the USA [30,105,106]. Two studies demonstrated SLEV infection of laboratory-reared *An. quadrimaculatus* [151] and *An. maculipennis* [176] (Table 2 and Table S3). Finally, USUV, an arbovirus that emerged in Italy in the 1990s, was detected three times in *An. maculipennis* from Italy [109,110,111] (Table S1).

Other arboviruses of the *Flavivirus* genus found in *Anopheles* included the Zika virus (ZIKV), detected five times; three of them in *An. sinensis* from China [93]. Of note, the Yellow Fever virus (YFV) was identified in *Anopheles neivai* during a Yellow Fever outbreak that occurred at the end of the 1940s in Panamá [137] (Table S1).

##### Other Arboviruses

After the *Peribunyaviridae*, *Togaviridae* and *Flaviviridae* families, the *Reoviridae* family is next for the greatest number of viruses detected in natural populations of *Anopheles*, with the Banna virus (BAV) and Liao ning virus (LNV) being the most detected (Table S1). BAV has been detected five times in *An. sinensis*, in Gansu, Yunnan, Yichang and Hubei provinces of China. Regarding LNV, it causes human encephalitis, and it is classified as a probable arbovirus; it was considered to be geographically limited to China, but it was later isolated from *Anopheles* populations in Australia on four occasions between 2014 and 2018. Among other arboviruses identified in *Anopheles*, the Rift Valley fever virus (RVFV) of the *Phenuiviridae* family was detected seven times in natural populations of *An. coustani*, *Anopheles squamosus* and *Anopheles arabiensis* in Africa. In laboratory studies, *An. stephensi* and *An. coustani* were susceptible to infection and had transmission capacity for RVFV [150,174] (Table 2 and Table S3).

#### 3.2.2. Insect-Specific Viruses (ISVs) Detected in *Anopheles*

**Figure 4:**
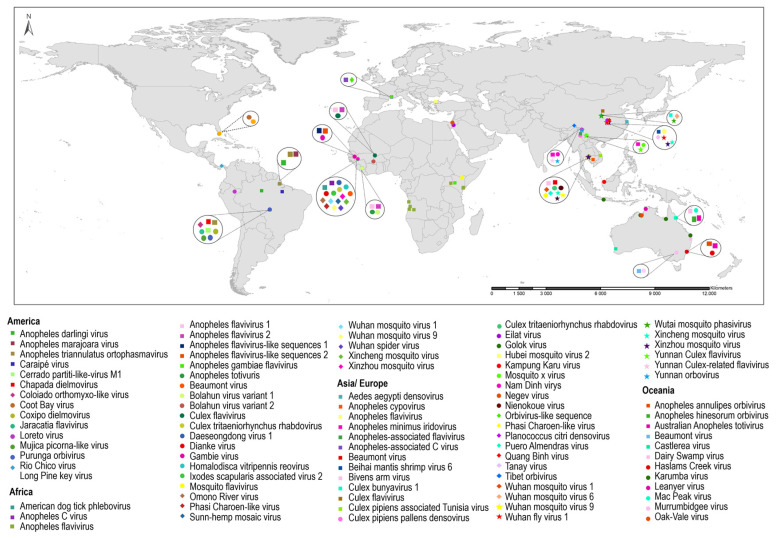
Worldwide distribution of Insect-Specific Viruses (ISVs) detected in wild-caught *Anopheles*.

Of the 84 ISV detections in *Anopheles* mosquitoes, 97% of reports were during the last 14 years (Figure 2b). The highest proportion of these ISVs belonged to the *Flaviviridae* and *Rhabdoviridae* families; however, for a large number of the more recently detected ISVs, their taxonomic classification at the family level was not possible (Figure 2a). The countries reporting the highest number of ISVs in *Anopheles* are China (21.8%), Senegal (17.6%), Australia (15.1%) and Brazil (10.9%) (Table 3 and Figure 4). The Anopheles flavivirus (AnFV) and its phylogenetically related variants, AnFV1 and AnFV2, are the ISVs more frequently detected in *Anopheles* natural populations, with 14 reports in the African and European continents (Table 3 and Figure 4). In particular, the Anopheles gambiae densovirus (AgDNV), a DNA virus of the *Parvoviridae* family isolated from the Sua5B cell line of *An. gambiae* is an attractive candidate for viral paratransgenesis in *Anopheles* mosquitoes [160]. This is due to features such as its capacity to infect various tissues of laboratory-reared *An. gambiae* larvae and adults and the establishment of a productive infection that is transmitted horizontally [159,160] (Table S3).

Other ISVs detected in *Anopheles* mosquitoes are Anopheles C virus (AnCV) and Anopheles cypovirus (AnCPV), both identified and isolated from natural populations of *An. gambiae* from Cambodia and Senegal [185] (Table S2). Under laboratory conditions, both viruses establish a productive infection and are transmitted transovarially in *Anopheles coluzzii* [158]. Similarly, the Dianke virus (DKV) was recently identified in natural populations of *An. funestus*, *An. gambiae*, *Anopheles pharoensis* and *Anopheles rufipes* from Senegal. DKV generates a productive infection in various tissues of *An. gambiae* [164]. Finally, in this review, no studies were found that identified Thai-strain densovirus (AThDNV) from *Anopheles* natural populations; however, a laboratory study indicated that this virus infects and is vertically transmitted in laboratory-reared *Anopheles minimus* [183] (Table S3).

**Table 3:** Most abundant Insect-Specific Viruses (ISVs) detected in wild-caught *Anopheles* mosquitoes worldwide.

Virus Name/Abbreviation	Country	*Anopheles* Species	References *
Anopheles flavivirus (AnFV)	Angola	*Anopheles* spp.	[186]
	Kenya	*An. gambiae*	[135]
		*An. gambiae* s.l.	[187]
		*An. squamosus*	[135]
	Turkey	*An. maculipennis* s.l.	[188]
Karumba virus (KRBV)	Australia	*An. meraukensis*	[101,189]
Dianke virus (DKV)	Senegal	*An. funestus*	[190]
		*An. gambiae*	[190]
		*An. pharoensis*	[190]
		*An. rufipes*	[190]
Xinzhou mosquito virus	Cambodia	*Anopheles* spp.	[116]
	China	*An. sinensis*	[191]
	Senegal	*Anopheles* spp.	[148]
Culex flavivirus (CxFV)	China	*An. sinensis*	[192]
	Guinea/Mali	*Anopheles* spp.	[193]
Beaumont virus	Australia	*An. annulipes* s.l.	[59]
	Cambodia	*Anopheles* spp.	[116]
	Senegal	*Anopheles* spp.	[116]
Xincheng mosquito virus	Cambodia	*Anopheles* spp.	[116]
	China	*An. sinensis*	[191]
	Senegal	*Anopheles* spp.	[116]
Tanay virus (TANAV)	China	*An. sinensis*	[89,194]
Hubei mosquito virus 2 (HMV2)	China	*An. sinensis*	[49,89]
Wuhan mosquito virus 1	Cambodia	*Anopheles* spp.	[116]
	Senegal	*Anopheles* spp.	[116]
Wuhan mosquito virus 9	Cambodia	*Anopheles* spp.	[116]
	Senegal	*Anopheles* spp.	[116]
Anopheles flavivirus 1 (AnFV1)	Guinea/Mali	*Anopheles* spp.	[193]
	Liberia	*An. gambiae*	[195]
Anopheles flavivirus 2 (AnFV2)	Guinea/Mali	*Anopheles* spp.	[193]
	Liberia	*An. gambiae*	[195]
Culex tritaeniorhynchus rhabdovirus	Cambodia	*Anopheles* spp.	[116]
	Senegal	*Anopheles* spp.	[116]
Anopheles minimus iridovirus (AMIV)	China	*An. minimus*	[50,196]

* Table S2 displays additional ISV that have been identified in wild-caught Anopheles [197,198,199,200,201,202,203,204,205,206,207,208,209,210,211,212,213,214].

#### 3.2.3. Other Viruses Detected in *Anopheles*

**Figure 5:**
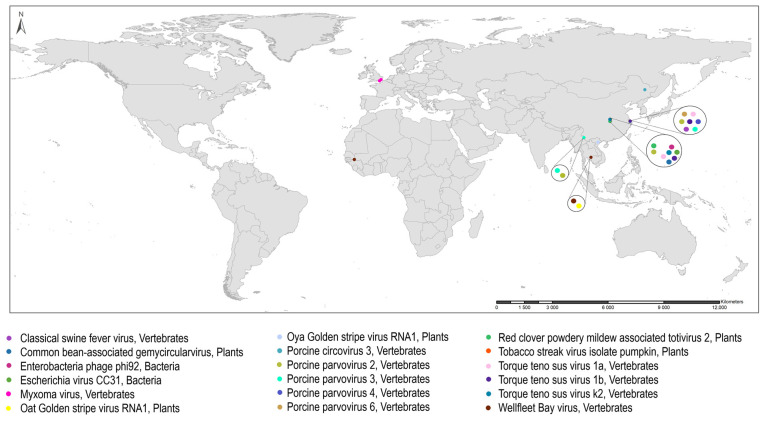
Worldwide distribution of viruses known to infect vertebrates, plants and prokaryotes detected in wild-caught *Anopheles*.

Although arboviruses and ISVs are the most frequently detected in *Anopheles* natural populations, this systematic review reports on other viruses known to infect vertebrates, plants or bacteria detected in *Anopheles* (Table 4 and Figure 5). Mosquitoes or other insects can act as mechanical vectors for some of the viruses that infect vertebrates. For example, Myxoma virus (MYXV), a virus that causes myxomatosis with the death of domestic rabbits, was reported twice in *Anopheles* mosquitoes from England; the first detection was in *Anopheles atroparvus* during an outbreak of myxomatosis in Newhaven County in 1954. Following this outbreak, a laboratory study demonstrated that members of a colony of semi-hibernating *An. atroparvus* can maintain MYXV infection up to 220 days post-infection and act as a mechanical vector of this virus [153] (Table S3). The other detection of MYXV was in specimens of the *An. maculipennis* complex collected while feeding on *Oryctolagus cuniculus* (European rabbit) in Kent County [215] (Table 2). Lastly, in recent studies, variants of the Porcine parvovirus (PPV), PPV2, PPV3, PPV4 and PPV6, were detected in *Anopheles* natural populations of China, most of them in *An. sinensis* (Table 4 and Figure 5).

## 4. Discussion

During this systematic review, 161 viruses detected in *Anopheles* natural populations worldwide were found, as well as 28 viruses infecting *Anopheles* in laboratory conditions. Thirty-five of the viruses detected in the natural *Anopheles* population are arboviruses, and twenty-four have been classified as probable arboviruses by the CDC’s International Catalog of Arboviruses [20]. Most of these studies have been conducted in *Anopheles* species of countries of Oceania, East and Southeast Asia, Europe and North America. The majority of studies and records of viruses detected in *Anopheles* are from the Global North, and fewer are from African, Latin American, Central and South Asian countries. Notably, this distribution coincides with the level of investment in science at a historical level in those countries [217].

For approximately eight decades, the methodologies or techniques used for viral detection in *Anopheles* have included cultured-dependent, immunological and molecular methods (Figure 2c); however, since the application of the Next Generation Sequence (NGS) methodologies, the number of viruses detected in *Anopheles* has increased exponentially [15,218] (Figure 2b,c). The use of NGS as a tool for viral detection evidenced that the utilization of animal models and cell cultures biased the reports towards the arboviral component, given that ISVs cannot be recovered in those systems [7]. In just a decade (2011–2021), NGS contributed to the detection of 97% of ISVs in *Anopheles*, which are the most abundant viral component in this mosquito population; in fact, they represent approximately ~52% of the total number of viruses reported in the scientific literature.

Most ISVs have been described in mosquitoes of the Culicidae family, mainly in the *Aedes*, *Culex* and *Anopheles* genera [219,220], known as Mosquito-specific viruses (MSVs). Of interest, some of the reported MSVs have the ability to generate a productive infection in their host and can be transmitted vertically or horizontally, as is the case of AgDNV, AnCV, AnCPV and DKV [164,185,221]. The ISVs have the potential to be used in biological control strategies against disease vectors; for example, AgDNV is a virus susceptible to genetic manipulation which could function as an expression vector in *Anopheles* mosquitoes on a viral paratransgenesis strategy [221].

The discovery of ISVs in *Anopheles* has also contributed to the field of evolutionary virology; for example, a close phylogenetic relationship has been established among some ISVs with medically relevant arboviruses [222]. As such, the Eilat Virus (EILV) of the *Togaviridae* family, isolated from *Anopheles coustani* in Egypt [223], is at the base of the phylogeny of the *Alphavirus* genus and is closely related to WEEV, although it is unable to infect vertebrate cells [224,225]. A similar relationship was found between ISVs of the *Bunyavirales* order and the *Flavivirus* genus [157,226]. These observations led to the hypothesis that arboviruses originated from ISVs circulating in mosquitoes and other vectors [223,224,227]. Moreover, some studies showed viral exclusion by superinfection of EILV and arboviruses of the *Alphavirus* genus in C7/10 cells of *Aedes albopictus*, given their genetic similarities [225]. In addition, because of the genetic similarities, EILV has been used as a platform for vaccine development against the WNV and EEEV viruses [228] and also as a model for the generation of antigens for the diagnosis of CHIKV in ELISA-type assays [229].

Furthermore, this review found 59 different arboviruses and probable arboviruses reported in *Anopheles* natural populations. Although their presence does not necessarily indicate that *Anopheles* is an arbovirus vector, various studies suggested that some *Anopheles* species could transmit arboviruses in addition to ONNV (Table 2). This assumption is supported by the following vector incrimination criteria: 1. Virus recovery from mosquito natural populations, 2. Evidence of mosquito contact with the vertebrate host, 3. Virus outbreaks and vector co-occurrence in space and time, and 4. Proof of virus transmission under laboratory conditions [155]. For example, RVFV was detected in the anthropophilic species *An. coustani* during outbreaks in Madagascar [74] and Sudan [12], and it was competent for RVFV transmission under laboratory conditions [150]. Similarly, the anthropophilic species *An. quadrimaculatus* was found infected with SLEV during an epidemic outbreak [106], and it was susceptible to SLEV infection under laboratory conditions [151]. Furthermore, various studies often reported arbovirus isolated during virus outbreaks (Table 2); conversely, there were no studies evidencing infection or transmission in *Anopheles* laboratory-reared mosquitoes by other arbovirus. As such, *An. subpictus* has been found with JEV in natural populations in India during JEV outbreaks [55]. In addition, JEV was detected in *An. subpictus* males, a possible indication of infection by transmission through transovarial/transovum or sexual route [54]. Also, WNV was detected in *An. punctipennis* in the USA and *An. maculipennis* in Romania and Serbia during WNV epidemic outbreaks [37,40].

In addition to arboviruses and ISVs, some studies detected specific viruses of vertebrates, plants and bacteria in *Anopheles*. Plant viruses detected in mosquitoes have been associated with acquisition through contact while resting on vegetation or during nectar feeding [230,231]. Their presence does not indicate that the mosquito is acting as their biological vector, but probably as a mechanical vector, facilitating their circulation in the ecosystems; though, the role of the mosquitoes in plant viruses spread has to be further explored [231,232,233].

Regarding the vertebrate-specific viruses detected in *Anopheles*, most are acquired by mosquito contact with host skin or during blood meal ingestion [144]. Some of these viruses are mechanically transmitted by vectors [231]; when a mosquito contaminates its mouthparts and head while in contact with a viremic host, it becomes able to transmit the virus to another host [234,235]. For instance, some works suggest that *Anopheles* can be a mechanical vector of vertebrate viruses such as MYXV, which was detected in *An. atroparvus* during an outbreak of myxomatosis in rabbits; also, *An. atroparvus* can maintain MYXV for up to 220 dpi and transmit it mechanically [154] (Table S3). Another virus, PPV, was repeatedly detected in *An. sinensis* from pig farms in China [49]. Although there is no evidence of mechanical transmission of PPV by mosquitoes, this virus can resist and survive on surfaces (e.g., metals, plastics, etc.), which enables its transmission to susceptible hosts [236], playing a role in their spread [237].

Finally, regarding the interaction of viruses with the *Plasmodium* parasite, few studies have addressed this subject. One study found that co-infection of RVFV and *Plasmodium* enhances the transmission of RVFV in *An. stephensi*. This is because *Plasmodium* disrupts the salivary gland barriers, facilitating the arbovirus passage [175]. Although virus-like particles have been detected in *Plasmodium* sporozoites [238], to date, there are no viruses infecting *Plasmodium* that have been characterized. Future works aimed to investigate the viruses harbored by both the parasite and host will contribute to elucidating trans-kingdom interactions among viruses, pathogens and mosquitoes; this research line has the potential to generate useful knowledge for the design of control strategies.

In conclusion, the knowledge of the viral component in *Anopheles* generated to date demonstrates the relevance of this topic for public health and basic science. The accelerated discovery of viruses associated with *Anopheles* in recent years has greatly contributed to the understanding of microbial community diversity virus–host relationships and has increased research on the potential practical applications of ISVs [7,221,222].

Despite these advances, more research on the viral component of *Anopheles* is needed, mostly when comparing the available information for other epidemiologically important mosquitoes such as *Aedes* and *Culex*. Also, a better understanding of the interaction dynamics between *Anopheles* and its arboviruses and their potential transmission is required. This is even more relevant in tropical regions where *Anopheles* is distributed, and arboviral diseases are often undiagnosed or confused with other febrile illnesses or malaria [6,239,240]. Furthermore, the *Anopheles* species with anthropophilic tendencies are constantly exposed to arboviruses during blood-feeding on humans and also on other vertebrates, possibly enabling the spread of viral pathogens. Finally, the accelerated anthropogenic alterations of wild environments are causing modifications in *Anopheles* species distribution, affecting the dynamics of disease transmission [241,242]. Altogether, this information reinforces the relevance of implementing the surveillance of viruses harbored and potentially transmitted by *Anopheles* mosquitoes, especially those of public health importance.

## Figures and Tables

**Table 1:** Most abundant arboviruses and probable arboviruses detected in wild-caught *Anopheles* mosquitoes worldwide.

Virus Name (Abbreviation)	Country	*Anopheles* Species	References *
Eastern equine encephalitis virus (EEE)	USA	*An. crucians*	[22,23,24,25]
		*An. crucians* complex	[26]
		*An. punctipennis*	[25,27,28]
		*An. quadrimaculatus*	[23,25,27,28,29]
Tensaw virus (TENV)	USA	*An. crucians*	[22,25,30,31,32,33,34]
		*An. crucians* complex	[26]
		*An. quadrimaculatus*	[22,31,32]
West Nile virus (WNV)	Israel	*An. coustani*	[35]
		*An. tenebrosus*	[36]
	Madagascar	*An. coustani*	[37]
		*An. pauliani*	[37,38]
	Romania	*An. hyrcanus*	[39]
		*An. maculipennis*	[39]
	Serbia	*An. maculipennis*	[40]
	Turkey	*An. claviger*	[41]
	USA	*An. atropos*	[42]
		*An. crucians*	[43]
		*An. franciscanus*	[44]
		*An. punctipennis*	[45,46,47]
		*An. quadrimaculatus*	[43]
		*An. walkeri*	[45]
Japanese encephalitis virus (JEV)	China	*An. sinensis*	[48,49,50,51]
	Philippines	*An. annularis*	[52]
	India	*An. barbirostris*	[14]
		*An. pallidus*	[14]
		*An. peditaeniatus*	[53]
		*An. subpictus*	[14,54,55]
	Indonesia	*An. annularis*	[56]
		*An. vagus*	[56]
	Malaysia	*Anopheles* spp.	[57]
	Taiwan	*An. sinensis*	[58]
Ross River virus (RRV)	Australia	*An. amictus*	[59,60,61]
		*An. annulipes*	[62]
		*An. annulipes* s.l.	[59]
		*An. bancroftii*	[61]
O’nyong-nyong virus (ONNV)	Democratic Republic of Congo	*Anopheles* spp.	[63]
	Kenya	*An. funestus*	[64,65]
		*An. gambiae*	[65]
	Uganda	*An. funestus*	[65,66]
		*An. gambiae*	[65]
Cache Valley virus (CVV)	Jamaica	*An. grabhami*	[67]
	USA	*An. punctipennis*	[68,69]
		*An. quadrimaculatus*	[68,69,70,71]
		*An. walkeri*	[69]
Rift Valley fever virus (RVFV)	Kenya	*An. squamosus*	[72]
		*Anopheles* spp.	[73]
	Madagascar	*An. coustani*	[74]
		*An. squamosus*	[74]
	Sudan	*An. arabiensis*	[12]
		*An. coustani*	[12]
Getah virus (GETV)	China	*An. sinensis*	[50,75,76,77,78]
	Malaysia	*Anopheles* spp.	[79]
	Russia	*An. hyrcanus*	[80]
Batai virus (BATV)	Germany	*An. daciae*	[81]
		*An. maculipennus* s.l.	[82]
		*An. messeae*	[81]
	Italy	*An. maculipennis*	[83,84]

* Table S1 displays additional arboviruses that have been identified in wild-caught Anopheles [85,86,87,88,89,90,91,92,93,94,95,96,97,98,99,100,101,102,103,104,105,106,107,108,109,110,111,112,113,114,115,116,117,118,119,120,121,122,123,124,125,126,127,128,129,130,131,132,133,134,135,136,137,138,139,140].

**Table 4:** Other viruses detected in wild-caught *Anopheles* mosquitoes worldwide.

Virus Name/ Abbreviation	Category	Country	*Anopheles* Species	References
Classical swine fever virus (CSFV)	Vertebrates	China	*Anopheles* spp.	[200]
Common bean-associated gemycircularvirus (CBaGmV)	Plants	China	*An. sinensis*	[89]
Enterobacteria phage phi92	Bacteria	China	*An. sinensis*	[89]
Escherichia virus CC31	Bacteria	China	*An. sinensis*	[89]
Myxoma virus (MYXV)	Vertebrates	England	*An. atroparvus*	[153]
			*An. maculipennis* s.l.	[215]
Oat golden stripe virus RNA1	Plants	Cambodia	*Anopheles* spp.	[116]
Oya virus (OYAV)	Vertebrates	Vietnam	*An. sinensis*	[107]
			*An. vagus*	[107]
Porcine circovirus 3 (PCV3)	Vertebrates	China	*An. sinensis*	[216]
Porcine parvovirus 2 (PPV2)	Vertebrates	China	*An. sinensis*	[49,89]
			*Anopheles* spp.	[200]
Porcine parvovirus 3 (PPV3)	Vertebrates	China	*An. sinensis*	[49]
			*Anopheles* spp.	[200]
Porcine parvovirus 4 (PPV4)	Vertebrates	China	*Anopheles* spp.	[200]
Porcine parvovirus 6 (PPV6)	Vertebrates	China	*Anopheles* spp.	[200]
Red clover powdery Mildew-associated totivirus 2	Plants	China	*An. sinensis*	[89]
Tobacco streak virus isolate pumpkin	Plants	Cambodia	*Anopheles* spp.	[116]
Torque teno sus virus 1a (TTSV)	Vertebrates	China	*An. sinensis*	[89]
			*Anopheles* spp.	[200]
Torque teno sus virus 1b (TTSV)	Vertebrates	China	*An. sinensis*	[89]
	Vertebrates		*Anopheles* spp.	[200]
Torque teno sus virus k2 (TTSV)	Vertebrates	China	*An. sinensis*	[89]
Wellfleet Bay virus (WBV)	Vertebrates	Cambodia	*Anopheles* spp.	[116]
	Vertebrates	Senegal	*Anopheles* spp.	[116]

## Data Availability

All relevant data are presented within the manuscript.

## Supplementary Materials

The following supporting information can be downloaded at https://www.mdpi.com/article/10.3390/tropicalmed8100459/s1. Table S1: Arboviruses and probable arbovirus detected in wild-caught *Anopheles* mosquitoes worldwide; Table S2: Insect-Specific Viruses (ISVs) detected in wild-caught *Anopheles* mosquitoes worldwide; Table S3: Summary of laboratory studies that evaluated virus infection and transmission in *Anopheles* mosquitoes.

## References

[1] Hay S.I., Guerra C.A., Tatem A.J., Noor A.M., Snow R.W. (2004). The Global Distribution and Population at Risk of Malaria: Past, Present, and Future. Lancet Infect. Dis..

[2] WHO (2021). World Malaria Report 2021. https://www.who.int/teams/global-malaria-programme/reports/world-malaria-report-2022.

[3] Manguin S., Bangs M.J., Pothikasikorn J., Chareonviriyaphap T. (2010). Review on Global Co-Transmission of Human *Plasmodium* Species and *Wuchereria bancrofti* by *Anopheles* Mosquitoes. Infect. Genet. Evol..

[4] Brault A.C., Foy B.D., Myles K.M., Kelly C.L.H., Higgs S., Weaver S.C., Olson K.E., Miller B.R., Powers A.M. (2004). Infection Patterns of O’Nyong Nyong Virus in the Malaria-Transmitting Mosquito, *Anopheles gambiae*. Insect Mol. Biol..

[5] Scott T.W., Takken W. (2012). Feeding Strategies of Anthropophilic Mosquitoes Result in Increased Risk of Pathogen Transmission. Trends Parasitol..

[6] Prasad N., Murdoch D.R., Reyburn H., Crump J.A. (2015). Etiology of Severe Febrile Illness in Low- and Middle-Income Countries: A Systematic Review. PLoS ONE.

[7] Bolling B.G., Weaver S.C., Tesh R.B., Vasilakis N. (2015). Insect-Specific Virus Discovery: Significance for the Arbovirus Community. Viruses.

[8] Tajudeen Y.A., Oladunjoye I.O., Mustapha M.O., Mustapha S.T., Ajide-Bamigboye N.T. (2021). Tackling the Global Health Threat of Arboviruses: An Appraisal of the Three Holistic Approaches to Health. Health Promot. Perspect..

[9] Chaves L.S.M., Fry J., Malik A., Geschke A., Sallum M.A.M., Lenzen M. (2020). Global Consumption and International Trade in Deforestation-Associated Commodities Could Influence Malaria Risk. Nat. Commun..

[10] Hernández-Valencia J.C., Rincón D.S., Marín A., Naranjo-Díaz N., Correa M.M. (2020). Effect of Land Cover and Landscape Fragmentation on Anopheline Mosquito Abundance and Diversity in an Important Colombian Malaria Endemic Region. PLoS ONE.

[11] Ellwanger J.H., Chies J.A.B. (2021). Zoonotic Spillover: Understanding Basic Aspects for Better Prevention. Genet. Mol. Biol..

[12] Seufi A.E.M., Galal F.H. (2010). Role of *Culex* and *Anopheles* Mosquito Species as Potential Vectors of Rift Valley Fever Virus in Sudan Outbreak, 2007. BMC Infect. Dis..

[13] Brustolin M., Pujhari S., Henderson C.A., Rasgon J.L. (2018). *Anopheles* Mosquitoes May Drive Invasion and Transmission of Mayaro Virus across Geographically Diverse Regions. PLoS Negl. Trop. Dis..

[14] Thenmozhi V., Balaji T., Venkatasubramani K., Dhananjeyan K., Selvam A., Rajamannar V., Tyagi B. (2016). Role of *Anopheles subpictus* Grassi in Japanese Encephalitis Virus Transmission in Tirunelveli, South India. Indian J. Med. Res..

[15] Greninger A.L. (2018). A Decade of RNA Virus Metagenomics Is (Not) Enough. Virus Res..

[16] Öhlund P., Lundén H., Blomström A.-L. (2019). Insect-Specific Virus Evolution and Potential Effects on Vector Competence. Virus Genes.

[17] Roundy C.M., Azar S.R., Rossi S.L., Weaver S.C., Vasilakis N., Kielian M., Mettenleiter T.C., Roossinck M.J. (2017). Chapter Four—Insect-Specific Viruses: A Historical Overview and Recent Developments. Advances in Virus Research.

[18] PRISMA Preferred Reporting Items for Systematic Reviews and Meta-Analyses (PRISMA). https://prisma-statement.org/.

[19] Ouzzani M., Hammady H., Fedorowicz Z., Elmagarmid A. (2016). Rayyan-a Web and Mobile App for Systematic Reviews. Syst. Rev..

[20] Centers for Disease Control and Prevention Arbovirus Catalog. https://wwwn.cdc.gov/arbocat/.

[21] Nanfack-Minkeu F., Vernick K.D. (2018). A Systematic Review of the Natural Virome of *Anopheles* Mosquitoes. Viruses.

[22] Chamberlain R.W., Sudia W.D., Coleman P.H., Johnston J.G., Work T.H. (1969). Arbovirus Isolations from Mosquitoes Collected in Waycross, Georgia, 1963, during an Outbreak of Equine Encephalitis. Am. J. Epidemiol..

[23] Cupp E.W., Tennessen K.J., Oldland W.K., Hassan H.K., Hill G.E., Katholi C.R., Unnasch T.R. (2004). Mosquito and Arbovirus Activity during 1997–2002 in a Wetland in Northeastern Mississippi. J. Med. Entomol..

[24] Day J., Stark L. (1996). Eastern Equine Encephalitis Transmission to Emus (*Dromaius novaehollandiae*) in Volusia County, Florida: 1992 through 1994. J. Am. Mosq. Control Assoc..

[25] Wozniak A., Dowda H.E., Tolson M.W., Karabatsos N., Vaughan D.R., Turner P.E., Ortiz D.I., Wills W. (2001). Arbovirus Surveillance in South Carolina, 1996–1998. J. Am. Mosq. Control Assoc..

[26] Ortiz D.I., Wozniak A., Tolson M.W., Turner P.E. (2005). Arbovirus Circulation, Temporal Distribution, and Abundance of Mosquito Species in Two Carolina Bay Habitats. Vector-Borne Zoonotic Dis..

[27] Oliver J., Lukacik G., Kokas J., Campbell S.R., Kramer L.D., Sherwood J.A., Howard J.J. (2018). Twenty Years of Surveillance for Eastern Equine Encephalitis Virus in Mosquitoes in New York State from 1993 to 2012. Parasit. Vectors.

[28] Oliver J.A., Tan Y., Haight J.D., Tober K.J., Gall W.K., Zink S.D., Kramer L.D., Campbell S.R., Howard J.J., Das S.R. (2020). Spatial and Temporal Expansions of Eastern Equine Encephalitis Virus and Phylogenetic Groups Isolated from Mosquitoes and Mammalian Cases in New York State from 2013 to 2019. Emerg. Microbes Infect..

[29] Bingham A.M., Burkett-Cadena N.D., Hassan H.K., McClure C.J.W., Unnasch T.R. (2014). Field Investigations of Winter Transmission of Eastern Equine Encephalitis Virus in Florida. Am. J. Trop. Med. Hyg..

[30] Bond J.O., Quick D.T., Witte J.J., Oard H.C. (1965). The 1962 Epidemic of St. Louis Encephalitis in Florida. Am. J. Epidemiol..

[31] Chamberlain R.W., Sudia W.D., Coleman P.H. (1969). Isolations of an Arbovirus of the Bunyamwera Group (Tensaw Virus) from Mosquitoes in the Southeastern United States, 1960–1963. Am. J. Trop. Med. Hyg..

[32] Chamberlain R.W., Sudia W.D., Work T.H., Coleman P.H., Newhouse V.F., Johnston J.G. (1969). Arbovirus Studies in South Florida, with Emphasis on Venezuelan Equine Encephalomyelitis Virus. Am. J. Epidemiol..

[33] Mitchell C.J., Morris C.D., Smith G.C., Karabatsos N., Vanlandingham D., Cody E. (1996). Arboviruses Associated with Mosquitoes from Nine Florida Counties during 1993. J. Am. Mosq. Control Assoc..

[34] Nayar J.K., Karabatsos N., Knight J.W., Godsey M., Chang J., Mitchell C.J. (2001). Mosquito Hosts of Arboviruses from Indian River County, Florida, during 1998. Fla. Entomol..

[35] Nir Y., Goldwasser R., Lasowski Y., Margalit J. (1968). Isolation of West Nile Virus Strains from Mosquitoes in Israel. Am. J. Epidemiol..

[36] Lustig Y., Hindiyeh M., Orshan L., Weiss L., Koren R., Katz-Likvornik S., Zadka H., Glatman-Freedman A., Mendelson E., Shulman L.M. (2016). Mosquito Surveillance for 15 Years Reveals High Genetic Diversity among West Nile Viruses in Israel. J. Infect. Dis..

[37] Maquart M., Boyer S., Rakotoharinome V.M., Ravaomanana J., Tantely M.L., Heraud J.M., Cardinale E. (2016). High Prevalence of West Nile Virus in Domestic Birds and Detection in 2 New Mosquito Species in Madagascar. PLoS ONE.

[38] Tantely L.M., Cêtre-Sossah C., Rakotondranaivo T., Cardinale E., Boyer S. (2017). Population Dynamics of Mosquito Species in a West Nile Virus Endemic Area in Madagascar. Parasite.

[39] Dinu S., Cotar A.I., Pănculescu-Gătej I.R., Fălcuţă E., Prioteasa F.L., Sîrbu A., Oprişan G., Bădescu D., Reiter P., Ceianu C.S. (2015). West Nile Virus Circulation in South-Eastern Romania, 2011 to 2013. Eurosurveillance.

[40] Kemenesi G., Krtinić B., Milankov V., Kutas A., Dallos B., Oldal M., Somogyi N., Németh V., Bányai K., Jakab F. (2014). West Nile Virus Surveillance in Mosquitoes, April to October 2013, Vojvodina Province, Serbia: Implications for the 2014 Season. Eurosurveillance.

[41] Reeves W.K., Miller M.M., Bayik O., Chapman L. (2017). Operational Mosquito and Vector-Borne Diseases Surveillance at Incirlik Air Base, Turkey. US Army Med. Dep. J..

[42] Hribar L.J., Vlach J.J., Demay D.J., Stark L.M., Stoner R.L., Godsey M.S., Burkhalter K.L., Spoto M.C., James S.S., Smith J.M. (2003). Mosquitoes Infected with West Nile Virus in the Florida Keys, Monroe County, Florida, USA. J. Med. Entomol..

[43] Unlu I., Kramer W.L., Roy A.F., Foil L.D. (2010). Detection of West Nile Virus RNA in Mosquitoes and Identification of Mosquito Blood Meals Collected at Alligator Farms in Louisiana. J. Med. Entomol..

[44] Pitzer J.B., Byford R.L., Vuong H.B., Steiner R.L., Creamer R.J., Caccamise D.F. (2009). Potential Vectors of West Nile Virus in a Semiarid Environment: Doa Ana County, New Mexico. J. Med. Entomol..

[45] Andreadis T.G., Anderson J.F., Vossbrinck C.R., Main A.J. (2004). Epidemiology of West Nile Virus in Connecticut: A Five-Year Analysis of Mosquito Data 1999–2003. Vector-Borne Zoonotic Dis..

[46] Kulasekera V.L., Kramer L., Nasci R.S., Mostashari F., Cherry B., Trock S.C., Glaser C., Miller J.R. (2001). West Nile Virus Infection in Mosquitoes, Birds, Horses, and Humans, Staten Island, New York, 2000. Emerg. Infect. Dis..

[47] Yaremych S.A., Warner R.E., Mankin P.C., Brawn J.D., Raim A., Novak R. (2004). West Nile Virus and High Death Rate in American Crows. Emerg. Infect. Dis..

[48] Zhang H., Zi D., Shi H., Mi Z., Gong Z., Zhang J., Hou Z. (1990). The Natural Infection Rate of Mosquitoes by Japanese Encephalitis B Virus in Yunnan Province, China. Chin. J. Prev. Med..

[49] Hameed M., Wahaab A., Shan T., Wang X., Khan S., Di D., Liu X., Zhang J.J., Anwar M.N., Nawaz M. (2021). A Metagenomic Analysis of Mosquito Virome Collected from Different Animal Farms at Yunnan–Myanmar Border of China. Front. Microbiol..

[50] Li L., Guo X., Zhao Q., Tong Y., Fan H., Sun Q., Xing S., Zhou H., Zhang J. (2017). Investigation on Mosquito-Borne Viruses at Lancang River and Nu River Watersheds in Southwestern China. Vector-Borne Zoonotic Dis..

[51] Liu H., Lu H.J., Liu Z.J., Jing J., Ren J.Q., Liu Y.Y., Lu F., Jin N.Y. (2013). Japanese Encephalitis Virus in Mosquitoes and Swine in Yunnan Province, China 2009–2010. Vector-Borne Zoonotic Dis..

[52] Ksiazek T.G., Trosper J.H., Cross J.H., Basaca-Sevilla V. (1980). Additional Isolations of Japanese Encephalitis Virus from the Philippines. Southeast Asian J. Trop. Med. Public Health.

[53] Mourya D.T., Ilkal M.A., Mishra A.C., Jacob P.G., Pant U., Ramanujam S., Mavale M.S., Bhat H.R., Dhanda V. (1989). Isolation of Japanese Encephalitis Virus from Mosquitoes Collected in Karnataka State, India from 1985 to 1987. Trans. R. Soc. Trop. Med. Hyg..

[54] Thenmozhi V., Rajendran R., Ayanar K., Manavalan R., Tyagi B.K. (2006). Long-Term Study of Japanese Encephalitis Virus Infection in *Anopheles subpictus* in Cuddalore District, Tamil Nadu, South India. Trop. Med. Int. Health.

[55] Dhanda V., Thenmozhi V., Kumar N.P., Hiriyan J., Arunachalam N., Balasubramanian A., Ilango A., Gajanana A. (1997). Virus Isolation from Wild-Caught Mosquitoes during a Japanese Encephalitis Outbreak in Kerala in 1996. Indian J. Med. Res..

[56] Olson J.G., Ksiazek T.G., Lee V.H., Tan R., Shope R.E. (1985). Isolation of Japanese Encephalitis Virus from *Anopheles annularis* and *Anopheles vagus* in Lombok, Indonesia. Trans. R. Soc. Trop. Med. Hyg..

[57] Simpson D.I.H., Bowen E.T.W., Platt G.S., Way H., Smith C.E.G., Peto S., Kamath S., Lim B.L., Lim T.W. (1970). Japanese Encephalitis in Sarawak: Virus Isolation and Serology in a Land Dyak Village. Trans. R. Soc. Trop. Med. Hyg..

[58] Su C.-L., Yang C.-F., Teng H.-J., Lu L.-C., Lin C., Tsai K.-H., Chen Y.-Y., Chen L.-Y., Chang S.-F., Shu P.-Y. (2014). Molecular Epidemiology of Japanese Encephalitis Virus in Mosquitoes in Taiwan during 2005–2012. PLoS Negl. Trop. Dis..

[59] Coffey L.L., Page B.L., Greninger A.L., Herring B.L., Russell R.C., Doggett S.L., Haniotis J., Wang C., Deng X., Delwart E.L. (2014). Enhanced Arbovirus Surveillance with Deep Sequencing: Identification of Novel *Rhabdoviruses* and *Bunyaviruses* in Australian Mosquitoes. Virology.

[60] Kay B.H., Hearnden M.N., Oliveira N.M.M., Sellner L.N., Hall R.A. (1996). *Alphavirus* Infection in Mosquitoes at the Ross River Reservoir, North Queensland, 1990–1993. J. Am. Mosq. Control Assoc..

[61] Van Den Hurk A.F., Nisbet D.J., Foley P.N., Ritchie S.A., Mackenzie J.S., Beebe N.W. (2002). Isolation of Arboviruses from Mosquitoes (Diptera: Culicidae) Collected from the Gulf Plains Region of Northwest Queensland, Australia. J. Med. Entomol..

[62] Azuolas J., Wishart E., Bibby S., Ainsworth C. (2003). Isolation of Ross River Virus from Mosquitoes and from Horses with Signs of Musculo-Skeletal Disease. Med. J. Aust..

[63] Mbanzulu K.M., Wumba R., Mukendi J.P.K., Zanga J.K., Shija F., Bobanga T.L., Aloni M.N., Misinzo G. (2017). Mosquito-Borne Viruses Circulating in Kinshasa, Democratic Republic of the Congo. Int. J. Infect. Dis..

[64] Johnson B.K., Gichogo A., Gitau G., Patel N., Ademba G., Highton R.B., Smith D.H. (1981). Recovery of O’Nyong-Nyong Virus from *Anopheles funestus* in Western Kenya. Trans. R. Soc. Trop. Med. Hyg..

[65] Williams M.C., Woodall J.P., Corbet P.S., Gillett J.D. (1965). O’Nyong-Nyong Fever: An Epidemic Virus Disease in East Africa VIII. Virus Isolations from *Anopheles* Mosquitoes. Trans. R. Soc. Trop. Med. Hyg..

[66] Lutwama J.J., Kayondo J., Savage H.M., Burkot T.R., Miller B.R. (1999). Epidemic O’Nyong-Nyong Fever in Southcentral Uganda, 1996–1997: Entomologic Studies in Bbaale Village, Rakai District. Am. J. Trop. Med. Hyg..

[67] Belle E.A., King S.D., Griffiths B.B., Grant L.S. (1980). Epidemiological Investigation for Arboviruses in Jamaica, West Indies. Am. J. Trop. Med. Hyg..

[68] Anderson J.F., Armstrong P.M., Misencik M.J., Bransfield A.B., Andreadis T.G., Molaei G. (2018). Seasonal Distribution, Blood-Feeding Habits, and Viruses of Mosquitoes in an Open-Faced Quarry in Connecticut, 2010 and 2011. J. Am. Mosq. Control Assoc..

[69] Andreadis T.G., Armstrong P.M., Anderson J.F., Main A.J. (2014). Spatial-Temporal Analysis of Cache Valley Virus (*Bunyaviridae: Orthobunyavirus*) Infection in Anopheline and Culicine Mosquitoes (Diptera: Culicidae) in the Northeastern United States, 1997–2012. Vector-Borne Zoonotic Dis..

[70] Kokernot R.H., Hayes J., Tempelis C.H., Chan D.H.M., Boyd K., Anderson R.J. (1969). Arbovirus Studies in the Ohio-Mississippi Basin, 1964–1967. Am. J. Trop. Med. Hyg..

[71] Kokernot R.H., Hayes J., Boyd K.R., Sullivan P.S. (1974). Arbovirus Studies in Houston, Texas, 1968–1970. J. Med. Entomol..

[72] Sang R., Kioko E., Lutomiah J., Warigia M., Ochieng C., O’Guinn M., Lee J.S., Koka H., Godsey M., Hoel D. (2010). Rift Valley Fever Virus Epidemic in Kenya, 2006/2007: The Entomologic Investigations. Am. J. Trop. Med. Hyg..

[73] LaBeaud A.D., Sutherland L.J., Muiruri S., Muchiri E.M., Gray L.R., Zimmerman P.A., Hise A.G., King C.H. (2011). Arbovirus Prevalence in Mosquitoes, Kenya. Emerg. Infect. Dis..

[74] Ratovonjato J., Olive M.M., Tantely L.M., Andrianaivolambo L., Tata E., Razainirina J., Jeanmaire E., Reynes J.M., Elissa N. (2011). Detection, Isolation, and Genetic Characterization of Rift Valley Fever Virus from *Anopheles (Anopheles) coustani, Anopheles (Anopheles) squamosus*, and *Culex (Culex) antennatus* of the Haute Matsiatra Region, Madagascar. Vector-Borne Zoonotic Dis..

[75] Fang Y., Zhang W., Xue J.B., Zhang Y. (2021). Monitoring Mosquito-Borne Arbovirus in Various Insect Regions in China in 2018. Front. Cell. Infect. Microbiol..

[76] Liu H., Zhang X., Li L.X., Shi N., Sun X.T., Liu Q., Jin N.Y., Si X.K. (2019). First Isolation and Characterization of Getah Virus from Cattle in Northeastern China. BMC Vet. Res..

[77] Sun X., Fu S., Gong Z., Ge J., Meng W., Feng Y., Wang J., Zhai Y., Wang H., Nasci R. (2009). Distribution of Arboviruses and Mosquitoes in Northwestern Yunnan Province, China. Vector-Borne Zoonotic Dis..

[78] Zhang H.L., Zhang Y.Z., Yang W.H., Feng Y., Nasci R.S., Yang J., Liu Y.H., Dong C.L., Li S., Zhang B.-S. (2013). Mosquitoes of Western Yunnan Province, China: Seasonal Abundance, Diversity, and Arbovirus Associations. PLoS ONE.

[79] Simpson D.I.H., Way H.J., Platt G.S., Bowen E.T.W., Hill M.N., Kamath S., Bendell P.J.E., Heathcote O.H.U. (1975). Arbovirus Infections in Sarawak, October 1968–February 1970: Getah Virus Isolations from Mosquitoes. Trans. R. Soc. Trop. Med. Hyg..

[80] Chumakov M.P., Moshkin A.V., Andreeva E.B. (1974). Isolation of Five Strains of Getah Virus from Mosquitoes in the Southern Part of the Amur Region, USSR. Tr. Instituta Polio. Virusn. Entsefalitov Akad. Meditsinskikh Nauk.

[81] Scheuch D., Schäfer M., Eiden M., Heym E., Ziegler U., Walther D., Schmidt-Chanasit J., Keller M., Groschup M., Kampen H. (2018). Detection of Usutu, Sindbis, and Batai Viruses in Mosquitoes (Diptera: Culicidae) Collected in Germany, 2011–2016. Viruses.

[82] Jöst H., Bialonski A., Schmetz C., Günther S., Becker N., Schmidt-Chanasit J. (2011). Short Report: Isolation and Phylogenetic Analysis of Batai Virus, Germany. Am. J. Trop. Med. Hyg..

[83] Calzolari M., Bonilauri P., Bellini R., Caimi M., Defilippo F., Maioli G., Albieri A., Medici A., Veronesi R., Pilani R. (2010). Arboviral Survey of Mosquitoes in Two Northern Italian Regions in 2007 and 2008. Vector-Borne Zoonotic Dis..

[84] Huhtamo E., Lambert A.J., Costantino S., Servino L., Krizmancic L., Boldorini R., Allegrini S., Grasso I., Korhonen E.M., Vapalahti O. (2013). Isolation and Full Genomic Characterization of Batai Virus from Mosquitoes, Italy 2009. J. Gen. Virol..

[85] Johansen C.A., Nisbet D.J., Zborowski P., Van Den Hurk A.F., Ritchie S.A., Mackenzie J.S. (2003). *Flavivirus* Isolations from Mosquitoes Collected from Western Cape York Peninsula, Australia, 1999–2000. J. Am. Mosq. Control Assoc..

[86] Liang G.D., Li L., Zhou G.L., Fu S.H., Li Q.P., Li F.S., He H.H., Jin Q., He Y., Chen B.Q. (2000). Isolation and Complete Nucleotide Sequence of a Chinese Sindbis-like Virus. J. Gen. Virol..

[87] Jöst H., Bialonski A., Storch V., Günther S., Becker N., Schmidt-Chanasit J. (2010). Isolation and Phylogenetic Analysis of Sindbis Viruses from Mosquitoes in Germany. J. Clin. Microbiol..

[88] Johnson B.K., Shockley P., Chanas A.C., Squires E.J., Gardner P., Wallace C., Simpson D.I.H., Bowen E.T.W., Platt G.S., Way H. (1977). Arbovirus Isolations from Mosquitoes: Kano Plain, Kenya. Trans. R. Soc. Trop. Med. Hyg..

[89] Xia H., Wang Y., Shi C., Atoni E., Zhao L., Yuan Z. (2018). Comparative Metagenomic Profiling of Viromes Associated with Four Common Mosquito Species in China. Virol. Sin..

[90] Liu H., Li M.H., Zhai Y.G., Meng W.S., Sun X.H., Cao Y.X., Fu S.H., Wang H.Y., Xu L.H., Tang Q. (2010). Banna Virus, China, 1987-2007. Emerg. Infect. Dis..

[91] Xia H., Liu H., Zhao L., Atoni E., Wang Y., Yuan Z. (2018). First Isolation and Characterization of a Group C Banna Virus (BAV) from *Anopheles sinensis* Mosquitoes in Hubei, China. Viruses.

[92] Barrio-Nuevo K.M., Cunha M.S., Luchs A., Fernandes A., Rocco I.M., Mucci L.F., DE Souza R.P., Medeiros-Sousa A.R., Ceretti-Junior W., Marrelli M.T. (2020). Detection of Zika and Dengue Viruses in Wildcaught Mosquitoes Collected during Field Surveillance in an Environmental Protection Area in São Paulo, Brazil. PLoS ONE.

[93] Wang J., Xu H., Song S., Cheng R., Fan N., Fu S., Zhang S., Xu Z., He Y., Lei W. (2021). Emergence of Zika Virus in *Culex tritaeniorhynchus* and *Anopheles sinensis* Mosquitoes in China. Virol. Sin..

[94] Diallo D., Sall A.A., Diagne C.T., Faye O., Faye O., Ba Y., Hanley K.A., Buenemann M., Weaver S.C., Diallo M. (2014). Zika Virus Emergence in Mosquitoes in Southeastern Senegal, 2011. PLoS ONE.

[95] Aspöck V.H., Kunz C. (1968). Isolierung Des Calovo-(=batai-=Chitoor-) Virus Aus Stechmücken in Österreich. Wien. Med. Wochensschr..

[96] Aspöck H., Kunz C. (1970). Überwinterung des Calovo-Virus in Experimentell Infizierten Weibchen von *Anopheles maculipennis* Messeae Fall. Bakteriol. Parasitenkd. Infekt. Hyg..

[97] Brudnjak Z., Danielova V., Ryba J., Vesenjak-Hirjan J. (1970). Isolation of Calovo Virus from *Anopheles maculipennis* s.l. Mosquitoes in Yugoslavia. Folia Parasitol..

[98] Danielová V., Málková D., Minár J., Rehse-Küpper B., Hájková Z., Halgos J., Jedlicka L. (1978). Arbovirus Isolations from Mosquitoes in South Slovakia. Folia Parasitol..

[99] Andreadis T.G., Anderson J.F., Armstrong P.M., Main A.J. (2008). Isolations of Jamestown Canyon Virus (*Bunyaviridae: Orthobunyavirus*) from Field-Collected Mosquitoes (Diptera: Culicidae) in Connecticut, USA: A Ten-Year Analysis, 1997–2006. Vector-Borne Zoonotic Dis..

[100] Heard P.B., Zhang M.B., Grimstad P.R. (1991). Laboratory Transmission of Jamestown Canyon and Snowshoe Hare Viruses (*Bunyaviridae*: California Serogroup) by Several Species of Mosquitoes. J. Am. Mosq. Control Assoc..

[101] Prow N.A., Mah M.G., Deerain J.M., Warrilow D., Colmant A.M.G., O’Brien C.A., Harrison J.J., McLean B.J., Hewlett E.K., Piyasena T.B.H. (2018). New Genotypes of Liao Ning Virus (LNV) in Australia Exhibit an Insect-Specific Phenotype. J. Gen. Virol..

[102] Cybinski D.H., Muller M.J. (1990). Isolation of Arboviruses from Cattle and Insects at Two Sentinel Sites in Queensland, Australia, 1979–1985. Aust. J. Zool..

[103] Standfast H., Dyce A., St George T.D., JMuller M., Doherty R., Carley J., Filippich C. (1984). Isolation of Arboviruses from Insects Collected at Beatrice Hill, Northern Territory of Australia, 1974–1976. Aust. J. Biol. Sci..

[104] Tzeng H.Y., Wu H.H., Ting L.J., Chang N.T., Chou Y.C., Tu W.C. (2019). Monitoring Taiwanese Bovine Arboviruses and Non-Arboviruses Using a Vector-Based Approach. Med. Vet. Entomol..

[105] Chamberlain R.W., Sudia W.D., Coleman P.H., Beadle L.D. (1964). Vector Studies in the St. Louis Encephalitis Tampa Bay Area, Florida, 1962. Am. J. Trop. Med. Hyg..

[106] Sudia W.D., Coleman P.H., Chamberlain R.W., Wiseman J.S., Work T.H. (1967). St. Louis Encephalitis Vector Studies in Houston, Texas, 1964. J. Med. Entomol..

[107] Bryant J.E., Crabtree M.B., Nam V.S., Yen N.T., Duc H.M., Miller B.R. (2005). Short Report: Isolation of Arboviruses from Mosquitoes Collected in Northern Vietnam. Am. J. Trop. Med. Hyg..

[108] Cao Y., Fu S., Song S., Cai L., Zhang H., Gao L., Cao L., Li M., Gao X., He Y. (2019). Isolation and Genome Phylogenetic Analysis of Arthropod-Borne Viruses, Including Akabane Virus, from Mosquitoes Collected in Hunan Province, China. Vector-Borne Zoonotic Dis..

[109] Calzolari M., Bonilauri P., Bellini R., Albieri A., Defilippo F., Tamba M., Tassinari M., Gelati A., Cordioli P., Angelini P. (2013). Usutu Virus Persistence and West Nile Virus Inactivity in the Emilia-Romagna Region (Italy) in 2011. PLoS ONE.

[110] Mancini G., Montarsi F., Calzolari M., Capelli G., Dottori M., Ravagnan S., Lelli D., Chiari M., Santilli A., Quaglia M. (2017). Specie di Zanzare Coinvolte Nella Circolazione dei Virus Della West Nile e Usutu in Italia. Vet. Ital..

[111] Verna F., Modesto P., Radaelli M.C., Francese D.R., Monaci E., Desiato R., Grattarola C., Peletto S., Mosca A., Savini G. (2017). Control of Mosquito-Borne Diseases in Northwestern Italy: Preparedness from One Season to the Next. Vector-Borne Zoonotic Dis..

[112] Doherty R.L., Carley J.G., Mackerras M.J., Marks E.N. (1963). Isolation and Characterization of Virus Strains from Wild-Caught Mosquitoes in North Queensland. Aust. J. Exp. Biol. Med. Sci..

[113] Ochieng C., Lutomiah J., Makio A., Koka H., Chepkorir E., Yalwala S., Mutisya J., Musila L., Khamadi S., Richardson J. (2013). Mosquito-Borne Arbovirus Surveillance at Selected Sites in Diverse Ecological Zones of Kenya; 2007–2012. Virol. J..

[114] Gordon S.W., Tammariello R.F., Linthicum K.J., Dohm D.J., Digoutte J.P., Calvo-Wilson M.A. (1992). Arbovirus Isolations from Mosquitoes Collected during 1988 in the Senegal River Basin. Am. J. Trop. Med. Hyg..

[115] Metselaar D., Kirya G.B., Geus A.D.E., Fever R.V., Sickness A.H. (1974). Isolation of Arboviruses in Kenya, 1966–1971. Trans. R. Soc. Trop. Med. Hyg..

[116] Belda E., Nanfack-Minkeu F., Eiglmeier K., Carissimo G., Holm I., Diallo M., Diallo D., Vantaux A., Kim S., Sharakhov I.V. (2019). De Novo Profiling of RNA Viruses in *Anopheles* Malaria Vector Mosquitoes from Forest Ecological Zones in Senegal and Cambodia. BMC Genom..

[117] Sudia W.D., Newhouse V.F., Chlisher C.H. (1975). Arbovirus Vector Ecology Studies in Mexico during the 1972 Venezuelan Equine Encephalitis Outbreak. Am. J. Epidemiol..

[118] Williams M.C., Woodall J.P., Corbet P.S. (1965). Nyando Virus: A Hitherto Undescribed Virus Isolated from *Anopheles funestus* Giles Collected in Kenya. Arch. Gesamte Virusforsch..

[119] Zhang W., Li F., Liu A., Lin X., Fu S., Song J., Liu G., Shao N., Tao Z., Wang Q. (2018). Identification and Genetic Analysis of Kadipiro Virus Isolated in Shandong Province, China. Virol. J..

[120] De Souza Lopes O., Forattini O.P., Fonseca I.E.M., Lacerda J.P.G., Sacchetta L.A., Rabello E.X. (1966). Boraceia Virus. A New Virus Related to Anopheles B Virus. EBM.

[121] De Souza Lopes O., De Abreu Sacchetta L. (1974). Epidemiology of Boraceia Virus in a Forested Area in São Paulo, Brazil. Am. J. Epidemiol..

[122] Bakhshi H., Mousson L., Moutailler S., Vazeille M., Piorkowski G., Zakeri S., Raz A., de Lamballerie X., Dinparast-Djadid N., Failloux A.B. (2020). Detection of Arboviruses in Mosquitoes: Evidence of Circulation of Chikungunya Virus in Iran. PLoS Negl. Trop. Dis..

[123] Diallo D., Fall G., Diagne C.T., Gaye A., Ba Y., Dia I., Faye O., Diallo M. (2020). Concurrent Amplification of Zika, Chikungunya, and Yellow Fever Virus in a Sylvatic Focus of Arboviruses in Southeastern Senegal, 2015. BMC Microbiol..

[124] Ajamma Y.U., Onchuru T.O., Ouso D.O., Omondi D., Masiga D.K., Villinger J. (2018). Vertical Transmission of Naturally Occurring Bunyamwera and Insect-Specific Flavivirus Infections in Mosquitoes from Islands and Mainland Shores of Lakes Victoria and Baringo in Kenya. PLoS Negl. Trop. Dis..

[125] Saluzzo J.F. (1983). Étude Écologique du Virus Orungo en Afrique Centrale. Ann. Inst. Pasteur Virol..

[126] Tomori A., Type O., Language T., Show M. (1976). Orungo (UgMP 359) Virus: A Hitherto Undescribed Virus, Biochemical, Biophysical and Epidemiological Studies. Ph.D. Thesis.

[127] Da Rosa J.F.S.T., de Andrade Travassos da Rosa A., Dégallier N., da Costa Vasconcelos P.F. (1992). Caracterização e Relacionamento Antigênico de Três Novos *Bunyavirus* No Grupo Anopheles A (*Bunyaviridae*) Dos Arbovirus. Rev. Saúde Pública.

[128] Batovska J., Buchmann J.P., Holmes E.C., Lynch S.E. (2020). Coding-Complete Genome Sequence of Yada Yada Virus, a Novel *Alphavirus* Detected in Australian Mosquitoes. Microbiol. Resour. Announc..

[129] Brown S.E., Gorman B.M., Tesh R.B., Knudson D.L. (1992). Isolation of Bluetongue and Epizootic Hemorrhagic Disease Viruses from Mosquitoes Collected in Indonesia. Vet. Microbiol..

[130] Simo Tchetgna H.D., Selekon B., Kazanji M., Berthet N., Nakoune E. (2019). Complete Genome Sequence of the Tataguine Virus, Isolated in the Central African Republic in 1972 from a Human with an Acute Febrile Syndrome. Microbiol. Resour. Announc..

[131] Cunha M.S., Luchs A., Da Costa A.C., Ribeiro G., Dos Santos F.C.P., Nogueira J.S., Komninakis S.V., dos Santos Souza Marinho R., Witkin S.S., Villanova F. (2020). Detection and Characterization of Ilheus and Iguape Virus Genomes in Historical Mosquito Samples from Southern Brazil. Acta Trop..

[132] Armstrong P.M., Andreadis T.G., Anderson J.F., Main A.J. (2005). Isolations of Potosi Virus from Mosquitoes (Diptera: Culicidae) Collected in Connecticut. J. Med. Entomol..

[133] Saluzzo J.F., Germain M., Huard M., Robin Y., Gonzalez J.-P., Herve J.-P., Georges A.-J., Heme G., Digoutte J.-P. (1983). Le Virus Bozo (ArB 7343): Un Nouvel Arbovirus Du Groupe Bunyamwera Isolé En République Centrafricaine; Sa Transmission Expérimentale Par *Aedes aegypti*. Ann. Inst. Pasteur Virol..

[134] Mitchell C.J., Monath T.P., Sabattini M.S., Cropp C.B., Daffner J.F., Calisher C.H., Jakob W.L., Christensen H.A. (1985). Arbovirus Investigations in Argentina, 1977–1980. II. Arthropod Collections and Virus Isolations from Argentine Mosquitoes. Am. J. Trop. Med. Hyg..

[135] Villinger J., Mbaya M.K., Ouso D., Kipanga P.N., Lutomiah J., Masiga D.K. (2017). Arbovirus and Insect-Specific Virus Discovery in Kenya by Novel Six Genera Multiplex High-Resolution Melting Analysis. Mol. Ecol. Resour..

[136] Rowley W.A., Wong Y.W., Dorsey D.C., Hausler W.J. (1973). Field Studies on Mosquito-Arbovirus Relationships in Iowa, 1971. J. Med. Entomol..

[137] De Rodaniche E., Galindo P., Johnson C.M. (1957). Isolation of Yellow Fever Virus from *Haemagogus lucifer*, *H. equinus*, *H. spegazzinii falco*, *Sabethes chloropterus* and *Anopheles neivai* Captured in Panama in the Fall of 1956. Am. J. Trop. Med. Hyg..

[138] Toi C.S., Webb C.E., Haniotis J., Clancy J., Doggett S.L. (2017). Seasonal Activity, Vector Relationships and Genetic Analysis of Mosquito-Borne Stratford Virus. PLoS ONE.

[139] Hubalek Z., Sebesta O., Pesko J., Betasova L., Blazejova H., Venclikova K., Rudolf I. (2014). Isolation of Tahyna Virus (California Encephalitis Group) from *Anopheles hyrcanus* (Diptera, Culicidae), a Mosquito Species New to, and Expanding in, Central Europe. J. Med. Entomol..

[140] Méndez-López M.R., Attoui H., Florin D., Calisher C.H., Florian-Carrillo J.C., Montero S. (2015). Association of Vectors and Environmental Conditions during the Emergence of Peruvian Horse Sickness Orbivirus and Yunnan Orbivirus in Northern Peru. J. Vector Ecol..

[141] Collins W., Harrison A. (1967). Studies of Tensaw Virus in *Anopheles quadrimaculatus, A. albimanus,* and *A. maculatus*. Mosq. News.

[142] Blackmore C.G.M., Blackmore M.S., Grimstad P.R. (1998). Role of *Anopheles quadrimaculatus* and *Coquillettidia perturbans* (Diptera: Culicidae) in the Transmission Cycle of Cache Valley Virus (*Bunyaviridae: Bunyavirus*) in the Midwest, U.S.A. J. Med. Entomol..

[143] Saliba E.K., DeFoliart G.R., Yuill T.M., Hanson R.P. (1973). Laboratory Transmission of Wisconsin Isolates of a Cache Valley like Virus by Mosquitoes. J. Med. Entomol..

[144] Odhiambo C., Venter M., Chepkorir E., Mbaika S., Lutomiah J., Swanepoel R., Sang R. (2014). Vector Competence of Selected Mosquito Species in Kenya for Ngari and Bunyamwera Viruses. J. Med. Entomol..

[145] Johnson B.K., Chanas A.C., Squires E.J., Shockley P., Simpson D.I.H., Smith D.H. (1978). The Isolation of a Bwamba Virus Variant from Man in Western Kenya. J. Med. Virol..

[146] Chanas A.C., Hubalek Z., Johnson B.K., Simpson D.I.H. (1979). A Comparative Study of O’Nyong Nyong Virus with Chikungunya Virus and Plaque Variants. Arch. Virol..

[147] Mumford J.D., Long C.A., Weaver S.C., Miura K., Wang E., Rotenberry R., Dotson E.M., Benedict M.Q. (2019). *Plasmodium falciparum* (Haemosporodia: Plasmodiidae) and O’Nyong-Nyong Virus Development in a Transgenic *Anopheles gambiae* (Diptera: Culicidae) Strain. J. Med. Entomol..

[148] Myles K.M., Kelly C.L.H., Ledermann J.P., Powers A.M. (2006). Effects of an Opal Termination Codon Preceding the NsP4 Gene Sequence in the O’Nyong-Nyong Virus Genome on *Anopheles gambiae* Infectivity. J. Virol..

[149] Vanlandingham D.L., Hong C., Klingler K., Tsetsarkin K., McElroy K.L., Powers A.M., Lehane M.J., Higgs S. (2005). Differential Infectivities of O’Nyong-Nyong and Chikungunya Virus Isolates in *Anopheles gambiae* and *Aedes aegypti* Mosquitoes. Am. J. Trop. Med. Hyg..

[150] Nepomichene T.N.J.J., Raharimalala F.N., Andriamandimby S.F., Ravalohery J.P., Failloux A.B., Heraud J.M., Boyer S. (2018). Vector Competence of *Culex antennatus* and *Anopheles coustani* Mosquitoes for Rift Valley Fever Virus in Madagascar. Med. Vet. Entomol..

[151] Webster L.T., Clow A.D., Bauer J.H. (1935). Experimental Studies on Encephalitis: III. Survival of Encephalitis Virus (St. Louis Type) in *Anopheles quadrimaculatus*. J. Exp. Med..

[152] Collins W., Harrison A.J., Jumper J.R. (1965). Infection and Transmission Studies with Eastern Encephalitis Virus and *Anopheles albimanus* and *A*. Quadrimaculatus. Mosq. News.

[153] Muirhead-Thomson R.C. (1956). Field Studies of the Role of *Anopheles atroparvus* in the Transmission of Myxomatosis in England. Epidemiol. Infect..

[154] Andrewes C.H., Muirhead-Thomson R.C., Stevenson J.P. (1956). Laboratory Studies of *Anopheles atroparvus* in Relation to Myxomatosis. J. Hyg..

[155] Weaver S.C. (2020). Incrimination of Mosquito Vectors. Nat. Microbiol..

[156] Ward T.W., Jenkins M.S., Afanasiev B.N., Edwards M., Duda B.A., Suchman E., Jacobs-Lorena M., Beaty B.J., Carlson J.O. (2001). *Aedes aegypti* Transducing Densovirus Pathogenesis and Expression in *Aedes aegypti* and *Anopheles gambiae* Larvae. Insect Mol. Biol..

[157] Cook S., Moureau G., Kitchen A., Gould E.A., de Lamballerie X., Holmes E.C., Harbach R.E. (2012). Molecular Evolution of the Insect-Specific Flaviviruses. J. Gen. Virol..

[158] Nanfack-Minkeu F., Mitri C., Bischoff E., Belda E., Casademont I., Vernick K.D. (2019). Interaction of RNA Viruses of the Natural Virome with the African Malaria Vector, *Anopheles coluzzii*. Sci. Rep..

[159] Barik T.K., Suzuki Y., Rasgon J.L. (2016). Factors Influencing Infection and Transmission of Anopheles gambiae Densovirus (AgDNV) in Mosquitoes. PeerJ.

[160] Ren X., Hoiczyk E., Rasgon J.L. (2008). Viral Paratransgenesis in the Malaria Vector *Anopheles gambiae*. PLoS Pathog..

[161] Hardy J.L., Lyness R.N., Rush W.A. (1972). Experimental Vector and Wildlife Host Ranges of Buttonwillow Virus in Kern County, California. Am. J. Trop. Med. Hyg..

[162] Gilotra S.K., Shah K.V. (1967). Laboratory Studies on Transmission of Chikungunya Virus by Mosquitoes. Am. J. Epidemiol..

[163] Yadav P., Gokhale M.D., Barde P.V., Singh D.K., Mishra A.C., Mourya D.T. (2003). Experimental Transmission of Chikungunya Virus by *Anopheles stephensi* Mosquitoes. Acta Virol..

[164] Gaye A., Diagne M.M., Ndiaye E.H., Dior Ndione M.H., Faye M., Talla C., Fall G., Ba Y., Diallo D., Dia I. (2020). Vector Competence of Anthropophilic Mosquitoes for a New Mesonivirus in Senegal. Emerg. Microbes Infect..

[165] Vaidyanathan R., Edman J.D., Cooper L.A., Scott T.W. (1997). Vector Competence of Mosquitoes (Diptera: Culicidae) from Massachusetts for a Sympatric Isolate of Eastern Equine Encephalomyelitis Virus. J. Med. Entomol..

[166] Nasar F., Haddow A.D., Tesh R.B., Weaver S.C. (2014). Eilat Virus Displays a Narrow Mosquito Vector Range. Parasit. Vectors.

[167] Dasgupta R., Cheng L.L., Bartholomay L.C., Christensen B.M. (2003). Flock House Virus Replicates and Expresses Green Fluorescent Protein in Mosquitoes. J. Gen. Virol..

[168] Dasgupta R., Free H.M., Zietlow S.L., Paskewitz S.M., Aksoy S., Lei S., Fuchs J., Changyun H., Christensen B.M. (2007). Replication of Flock House Virus in Three Genera of Medically Important Insects. J. Med. Entomol..

[169] Hirumi H., Burton G.J., Maramorosch K. (1971). Electron Microscopy of Friend Murine Leukemia Virus in the Mid-Gut of Experimentally Infected Mosquitoes. J. Virol..

[170] Blow J.A., Turell M.J., Walker E.D., Silverman A.L. (2002). Post-Bloodmeal Diuretic Shedding of Hepatitis B Virus by Mosquitoes (Diptera: Culicidae). J. Med. Entomol..

[171] Dieme C., Ciota A.T., Kramer L.D. (2020). Transmission Potential of Mayaro Virus by *Aedes albopictus*, and *Anopheles quadrimaculatus* from the USA. Parasit. Vectors.

[172] Kramer L.D., Hardy J.L., Reeves W.C., Presser S.B., Bowen M.D., Eldridge B.F. (1993). Vector Competence of Selected Mosquito Species (Diptera: Culicidae) for California Strains of Northway Virus (*Bunyaviridae: Bunyavirus*). J. Med. Entomol..

[173] Gargan T.P., Clark G.G., Dohm D.J., Turell M.J., Bailey C.L. (1988). Vector Potential of Selected North American Mosquito Species for Rift Valley Fever Virus. Am. J. Trop. Med. Hyg..

[174] Turell M.J., Romoser W.S. (1994). Effect of the Developmental Stage at Infection on the Ability of Adult *Anopheles stephensi* to Transmit Rift Valley Fever Virus. Am. J. Trop. Med. Hyg..

[175] Vaughan J.A., Turell M.J. (1996). Facilitation of Rift Valley Fever Virus Transmission by *Plasmodium berghei* Sporozoites in *Anopheles stephensi* Mosquitoes. Am. J. Trop. Med. Hyg..

[176] Hammon W.M., Reeves W.C. (1943). Laboratory Transmission of St. Louis Encephalitis Virus by Three Genera of Mosquitoes. J. Exp. Med..

[177] Collins W. (1963). Transmission of Semliki Forest Virus by *Anopheles albimanus* Using Membrane Feeding Techniques. Mosq. News.

[178] Collins W., Harrison A.J., Skinner J.C. (1964). The Use of a Membrane Feeding Technique to Determine Infection and Transmission Thresholds of Semliki Forest Virus in *Anopheles quadrimaculatus* and *Anopheles albimanus*. Mosq. News.

[179] Collins W., Harrison A.J., Skinner J.C. (1965). Studies on the Transmission of Semliki Forest Virus by *Anopheles freeborni, A. stephensi, A. labranchiae atroparvus* and *A. sundaicus*. Mosq. News.

[180] Collins W., Harrison A.J. (1966). Studies of Sindbis Virus in *Anopheles albimanus* and *Aedes aegypti*. Mosq. News.

[181] Stollar V., Hardy J.L. (1984). Host-Dependent Mutants of Sindbis Virus Whose Growth Is Restricted in Cultured *Aedes albopictus* Cells Produce Normal Yields of Virus in Intact Mosquitoes. Virology.

[182] Ledermann J.P., Zeidner N., Borland E.M., Mutebi J.P., Lanciotti R.S., Miller B.R., Lutwama J.J., Tendo J.M., Andama V., Powers A.M. (2014). Sunguru Virus: A Novel Virus in the Family *Rhabdoviridae* Isolated from a Chicken in North-Western Uganda. J. Gen. Virol..

[183] Rwegoshora R.T., Kittayapong P. (2004). Pathogenicity and Infectivity of the Thai-Strain Densovirus (AThDNV) in *Anopheles minimus* s.l. Southeast Asian J. Trop. Med. Public Health.

[184] Bautista Garfias C.R., Mercado Sanchez S., Morilla González A. (1977). Experimental Infection of *Anopheles albimanus* and *Culex thriambus* Mosquitoes with Venezuelan Equine Encephalomyelitis Virus TC-83 Strain. Mosq. News.

[185] Carissimo G., Eiglmeier K., Reveillaud J., Holm I., Diallo M., Diallo D., Vantaux A., Kim S., Ménard D., Siv S. (2016). Identification and Characterization of Two Novel RNA Viruses from *Anopheles gambiae* Species Complex Mosquitoes. PLoS ONE.

[186] Morais P., Pinto J., Jorge C.P., Troco A.D., Fortes F., Sousa C.A., Parreira R. (2020). Insect-Specific Flaviviruses and Densoviruses, Suggested to Have Been Transmitted Vertically, Found in Mosquitoes Collected in Angola: Genome Detection and Phylogenetic Characterization of Viral Sequences. Infect. Genet. Evol..

[187] Iwashita H., Higa Y., Futami K., Lutiali P.A., Njenga S.M., Nabeshima T., Minakawa N. (2018). Mosquito Arbovirus Survey in Selected Areas of Kenya: Detection of Insect-Specific Virus. Trop. Med. Health.

[188] Öncü C., Brinkmann A., Günay F., Kar S., Öter K., Sarıkaya Y., Nitsche A., Linton Y.-M., Alten B., Ergünay K. (2018). West Nile Virus, Anopheles flavivirus, a Novel Flavivirus as Well as Merida-like Rhabdovirus Turkey in Field-Collected Mosquitoes from Thrace and Anatolia. Infect. Genet. Evol..

[189] Colmant A.M.G., Hobson-Peters J., Bielefeldt-Ohmann H., van den Hurk A.F., Hall-Mendelin S., Chow W.K., Johansen C.A., Fros J., Simmonds P., Watterson D. (2017). A New Clade of Insect-Specific Flaviviruses from Australian *Anopheles* Mosquitoes Displays Species-Specific Host Restriction. mSphere.

[190] Diagne M.M., Gaye A., Ndione M.H.D., Faye M., Fall G., Dieng I., Widen S.G., Wood T.G., Popov V., Guzman H. (2020). Dianke Virus: A New Mesonivirus Species Isolated from Mosquitoes in Eastern Senegal. Virus Res..

[191] Li C.X., Shi M., Tian J.H., Lin X.D., Kang Y.J., Chen L.J., Qin X.C., Xu J., Holmes E.C., Zhang Y.Z. (2015). Unprecedented Genomic Diversity of RNA Viruses in Arthropods Reveals the Ancestry of Negative-Sense RNA Viruses. eLife.

[192] Liang W., He X., Liu G., Zhang S., Fu S., Wang M., Chen W., He Y., Tao X., Jiang H. (2015). Distribution and Phylogenetic Analysis of Culex flavivirus in Mosquitoes in China. Arch. Virol..

[193] Cannon M.V., Bogale H.N., Bhalerao D., Keita K., Camara D., Barry Y., Keita M., Coulibaly D., Kone A.K., Doumbo O.K. (2021). High-Throughput Detection of Eukaryotic Parasites and Arboviruses in Mosquitoes. Biol. Open.

[194] Zhao L., Mwaliko C., Atoni E., Wang Y., Zhang Y., Zhan J., Hu X., Xia H., Yuan Z. (2019). Characterization of a Novel Tanay Virus Isolated from *Anopheles sinensis* Mosquitoes in Yunnan, China. Front. Microbiol..

[195] Fauver J.R., Grubaugh N.D., Krajacich B.J., Weger-Lucarelli J., Lakin S.M., Fakoli L.S., Bolay F.K., Diclaro J.W., Dabiré K.R., Foy B.D. (2016). West African *Anopheles gambiae* Mosquitoes Harbor a Taxonomically Diverse Virome Including New Insect-Specific Flaviviruses, Mononegaviruses, and Totiviruses. Virology.

[196] Huang Y., Li S., Zhao Q., Pei G., An X., Guo X., Zhou H., Zhang Z., Zhang J., Tong Y. (2015). Isolation and Characterization of a Novel Invertebrate Iridovirus from Adult *Anopheles minimus* (AMIV) in China. J. Invertebr. Pathol..

[197] Da Silva Neves N.A., Pinto A.Z.L., Melo F.L., Maia L.M.S., da Silva Ferreira R., de Carvalho M.S., de Campos Júnior F.A., Nunes M.R.T., Ribeiro B.M., Slhessarenko R.D. (2021). Sialovirome of Brazilian Tropical Anophelines. Virus Res..

[198] Scarpassa V.M., Debat H.J., Alencar R.B., Saraiva J.F., Calvo E., Arcà B., Ribeiro J.M.C. (2019). An Insight into the Sialotranscriptome and Virome of Amazonian Anophelines. BMC Genom..

[199] Colmant A.M.G., Etebari K., Webb C.E., Ritchie S.A., Jansen C.C., van den Hurk A.F., Bielefeldt-Ohmann H., Hobson-Peters J., Asgari S., Hall R.A. (2017). Discovery of New Orbiviruses and Totivirus from *Anopheles* Mosquitoes in Eastern Australia. Arch. Virol..

[200] Hameed M., Liu K., Anwar M.N., Wahaab A., Li C., Di D., Wang X., Khan S., Xu J., Li B. (2019). A Viral Metagenomic Analysis Reveals Rich Viral Abundance and Diversity in Mosquitoes from Pig Farms. Transbound. Emerg. Dis..

[201] Fang Y., Li X.S., Zhang W., Xue J.B., Wang J.Z., Yin S.Q., Li S.G., Li X.H., Zhang Y. (2021). Molecular Epidemiology of Mosquito-Borne Viruses at the China–Myanmar Border: Discovery of a Potential Epidemic Focus of Japanese Encephalitis. Infect. Dis. Poverty.

[202] Cook S., Chung B.Y.W., Bass D., Moureau G., Tang S., McAlister E., Culverwell C.L., Glücksman E., Wang H., Brown T.D.K. (2013). Novel Virus Discovery and Genome Reconstruction from Field Rna Samples Reveals Highly Divergent Viruses in Dipteran Hosts. PLoS ONE.

[203] O’Brien C.A., McLean B.J., Colmant A.M.G., Harrison J.J., Hall-Mendelin S., van den Hurk A.F., Johansen C.A., Watterson D., Bielefeldt-Ohmann H., Newton N.D. (2017). Discovery and Characterisation of Castlerea Virus, a New Species of *Negevirus* Isolated in Australia. Evol. Bioinform..

[204] Contreras M.A., Eastwood G., Guzman H., Popov V., Savit C., Uribe S., Kramer L.D., Wood T.G., Widen S.G., Fish D. (2017). Almendravirus: A Proposed New Genus of Rhabdoviruses Isolated from Mosquitoes in Tropical Regions of the Americas. Am. J. Trop. Med. Hyg..

[205] He X., Yin Q., Zhou L., Meng L., Hu W., Li F., Li Y., Han K., Zhang S., Fu S. (2021). Metagenomic Sequencing Reveals Viral Abundance and Diversity in Mosquitoes from the Shaanxi-Gansu-Ningxia Region, China. PLoS Negl. Trop. Dis..

[206] Debat H.J., Ribeiro J.M. (2018). A Divergent Strain of *Culex pipiens*-Associated Tunisia Virus in the Malaria Vector *Anopheles epiroticus*. Microbiol. Resour. Announc..

[207] Samina I., Margalit J., Peleg J. (1986). Isolation of Viruses from Mosquitoes of the Negev, Israel. Trans. R. Soc. Trop. Med. Hyg..

[208] Guzman H., Contreras-Gutierrez M.A., Travassos da Rosa A.P.A., Nunes M.R.T., Cardoso J.F., Popov V.L., Young K.I., Savit C., Wood T.G., Widen S.G. (2018). Characterization of Three New Insect-Specific Flaviviruses: Their Relationship to the Mosquito-Borne Flavivirus Pathogens. Am. J. Trop. Med. Hyg..

[209] Doherty R.L., Carley J.G., Filippich C., Kay B.H., Gorman B.M., Rajapaksa N. (1977). Isolation of Sindbis (*Alphavirus*) and Leanyer Viruses from Mosquitoes Collected in the Northern Territory of Australia, 1974. Aust. J. Exp. Biol. Med. Sci..

[210] Vasilakis N., Forrester N.L., Palacios G., Nasar F., Savji N., Rossi S.L., Guzman H., Wood T.G., Popov V., Gorchakov R. (2013). Negevirus: A Proposed New Taxon of Insect-Specific Viruses with Wide Geographic Distribution. J. Virol..

[211] Huang Y., Mi Z., Zhuang L., Ma M., An X., Liu W., Cao W., Tong Y. (2013). Presence of Entomobirnaviruses in Chinese Mosquitoes in the Absence of Dengue Virus Coinfection. J. Gen. Virol..

[212] Quan P.-L., Williams D.T., Johansen C.A., Jain K., Petrosov A., Diviney S.M., Tashmukhamedova A., Hutchison S.K., Tesh R.B., Mackenzie J.S. (2011). Genetic Characterization of K13965, a Strain of Oak Vale Virus from Western Australia. Virus Res..

[213] Li M., Zheng Y., Zhao G., Fu S., Wang D., Wang Z., Liang G. (2014). Tibet Orbivirus, a Novel Orbivirus Species Isolated from *Anopheles maculatus* Mosquitoes in Tibet, China. PLoS ONE.

[214] Zuo S., Zhao Q., Guo X., Zhou H., Cao W., Zhang J. (2014). Detection of Quang Binh Virus from Mosquitoes in China. Virus Res..

[215] Brugman V.A., Hernández-Triana L.M., Prosser S.W.J., Weland C., Westcott D.G., Fooks A.R., Johnson N. (2015). Molecular Species Identification, Host Preference and Detection of Myxoma Virus in the *Anopheles maculipennis* Complex (Diptera: Culicidae) in Southern England, UK. Parasit. Vectors.

[216] Ha Z., Li J.F., Xie C.Z., Li C.H., Zhou H.N., Zhang Y., Hao P.F., Nan F.L., Zhang J.Y., Han J.C. (2020). First Detection and Genomic Characterization of Porcine Circovirus 3 in Mosquitoes from Pig Farms in China. Vet. Microbiol..

[217] Organisation for Economic Co-operation and Development (OECD) Gross Domestic Spending on Total, % of GDP 2000 – 2022, Annual 2022. https://data.oecd.org/chart/7bTc.

[218] National Research Council (US) Committee on Metagenomics (2017). The New Science of Metagenomics: Revealing the Secrets of Our Microbial Planet.

[219] De Almeida J.P.P., Aguiar E.R.G.R., Armache J.N., Olmo R.P., Marques J.T. (2021). The Virome of Vector Mosquitoes. Curr. Opin. Virol..

[220] Moonen J.P., Schinkel M., van der Most T., Miesen P., van Rij R.P. (2023). Composition and Global Distribution of the Mosquito Virome—A Comprehensive Database of Insect-Specific Viruses. One Health.

[221] Ren X., Rasgon J.L. (2010). Potential for the Anopheles gambiae Densonucleosis Virus to Act as an “Evolution-Proof” Biopesticide. J. Virol..

[222] Carvalho V.L., Long M.T. (2021). Insect-Specific Viruses: An Overview and Their Relationship to Arboviruses of Concern to Humans and Animals. Virology.

[223] Nasar F., Palacios G., Gorchakov R.V., Guzman H., Da Rosa A.P.T., Savji N., Popov V.L., Sherman M.B., Lipkin W.I., Tesh R.B. (2012). Eilat Virus, a Unique *Alphavirus* with Host Range Restricted to Insects by RNA Replication. Proc. Natl. Acad. Sci. USA.

[224] Hermanns K., Zirkel F., Kopp A., Marklewitz M., Rwego I.B., Estrada A., Gillespie T.R., Drosten C., Junglen S. (2017). Discovery of a Novel *Alphavirus* Related to Eilat Virus. J. Gen. Virol..

[225] Nasar F., Erasmus J.H., Haddow A.D., Tesh R.B., Weaver S.C. (2015). Eilat Virus Induces Both Homologous and Heterologous Interference. Virology.

[226] Ballinger M.J., Bruenn J.A., Hay J., Czechowski D., Taylor D.J. (2014). Discovery and Evolution of Bunyavirids in Arctic Phantom Midges and Ancient Bunyavirid-like Sequences in Insect Genomes. J. Virol..

[227] Hanley K.A., Weaver S.C., Domingo E., Parrish C.R., Holland J.J. (2008). CHAPTER 16—Arbovirus Evolution. Origin and Evolution of Viruses.

[228] Erasmus J.H., Seymour R.L., Kaelber J.T., Kim D.Y., Leal G., Sherman M.B., Frolov I., Chiu W., Weaver S.C., Nasar F. (2018). Novel Insect-Specific Eilat Virus-Based Chimeric Vaccine Candidates Provide Durable, Mono- and Multivalent, Single-Dose Protection against Lethal Alphavirus Challenge. J. Virol..

[229] Erasmus J.H., Needham J., Raychaudhuri S., Diamond M.S., Beasley D.W.C., Morkowski S., Salje H., Fernandez Salas I., Kim D.Y., Frolov I. (2015). Utilization of an Eilat Virus-Based Chimera for Serological Detection of Chikungunya Infection. PLoS Negl. Trop. Dis..

[230] Öhlund P., Hayer J., Lundén H., Hesson J.C., Blomström A.-L. (2019). Viromics Reveal a Number of Novel RNA Viruses in Swedish Mosquitoes. Viruses.

[231] Gray S.M., Banerjee N. (1999). Mechanisms of Arthropod Transmission of Plant and Animal Viruses. Microbiol. Mol. Biol. Rev..

[232] Dietzgen R.G., Mann K.S., Johnson K.N. (2016). Plant Virus-Insect Vector Interactions: Current and Potential Future Research Directions. Viruses.

[233] Ng T.F.F., Willner D.L., Lim Y.W., Schmieder R., Chau B., Nilsson C., Anthony S., Ruan Y., Rohwer F., Breitbart M. (2011). Broad Surveys of DNA Viral Diversity Obtained through Viral Metagenomics of Mosquitoes. PLoS ONE.

[234] Chamberlain R.W., Sudia W.D. (1961). Mechanism of Transmission of Viruses by Mosquitoes. Annu. Rev. Entomol..

[235] Chihota C.M., Rennie L.F., Kitching R.P., Mellor P.S. (2001). Mechanical Transmission of Lumpy Skin Disease Virus by *Aedes aegypti* (Diptera: Culicidae). Epidemiol. Infect..

[236] Eterpi M., McDonnell G., Thomas V. (2009). Disinfection Efficacy against Parvoviruses Compared with Reference Viruses. J. Hosp. Infect..

[237] Bagshaw C., Isdell A.E., Thiruvaiyaru D.S., Brisbin I.L., Sanchez S. (2014). Molecular Detection of Canine Parvovirus in Flies (Diptera) at Open and Closed Canine Facilities in the Eastern United States. Prev. Vet. Med..

[238] Garnham P.C., Bird R.G., Baker J.R. (1962). Electron Microscope Studies of Motile Stages of Malaria Parasites. III The Ookinetes of *Haemamoeba* and *Plasmodium*. Trans. R. Soc. Trop. Med. Hyg..

[239] Kaya A., Ergul N., Kaya S.Y., Kilic F., Yilmaz M.H., Besirli K., Ozaras R. (2013). The Management and the Diagnosis of Fever of Unknown Origin. Expert Rev. Anti-Infect. Ther..

[240] Odaga J., Sinclair D., Lokong J.A., Donegan S., Hopkins H., Garner P. (2014). Rapid Diagnostic Tests versus Clinical Diagnosis for Managing People with Fever in Malaria Endemic Settings. Cochrane Database Syst. Rev..

[241] Afrane Y.A., Githeko A.K., Yan G. (2012). The Ecology of *Anopheles* Mosquitoes under Climate Change: Case Studies from the Effects of Deforestation in East African Highlands. Ann. N. Y. Acad. Sci..

[242] Hertig E. (2019). Distribution of *Anopheles* Vectors and Potential Malaria Transmission Stability in Europe and the Mediterranean Area under Future Climate Change. Parasit. Vectors.

