# Supplementary material for: A Systematic Review on the Viruses of Anopheles Mosquitoes: The Potential Importance for Public Health
Source: Trop Med Infect Dis. 2023 Sep 26;8(10):459. doi: 10.3390/tropicalmed8100459 (PMC10610971; doi:10.3390/tropicalmed8100459)
Supplement: Supplementary file 1 [file tropicalmed-08-00459-s001.zip › tropicalmed-2602109-supplementary.pdf]

Supplementary material

Table S1. Arboviruses and probable arbovirus detected in wild-caught *Anopheles* mosquitoes worldwide.

Virus name/ abbreviation	Country	*Anopheles* species	References
Eastern equine encephalitis virus (EEEV)	USA	*An. crucians*	[22–25]
		An. crucians complex	[26]
		An. punctipennis	[25,27,28]
		An. quadrimaculatus	[23,25,27–29]
Tensaw virus (TENV)	USA	*An. crucians*	[22,25,30–34]
		An. crucians complex	[26]
		An. quadrimaculatus	[22,31,32]
West Nile virus (WNV)	Israel	*An. coustani*	[35]
		An. tenebrosus	[36]
	Madagascar	*An. coustani*	[37]
		An. pauliani	[37,38]
	Romania	*An. hyrcanus*	[39]
		An. maculipennis	[39]
	Serbia	*An. maculipennis*	[40]
	Turkey	*An. claviger*	[41]
	USA	*An. atropos*	[42]
		An. crucians	[43]
		An. franciscanus	[44]
		An. punctipennis	[45–47]
		An. quadrimaculatus	[43]
		An. walkeri	[45]
Japanese encephalitis virus (JEV)	China	*An. sinensis*	[48–51]
	Philippines	*An. annularis*	[52]
	India	*An. barbirostris*	[14]
		An. pallidus	[14]
		An. peditaeniatus	[53]
		An. subpictus	[14,54,55]
	Indonesia	*An. annularis*	[56]
		An. vagus	[56]
	Malaysia	*Anopheles* spp.	[57]
	Taiwan	*An. sinensis*	[58]
Ross River virus (RRV)	Australia	*An. amictus*	[59–61]
		An. annulipes	[62]
		An. annulipes s.l.	[59]
		An. bancroftii	[61]

O'nyong'nyong virus (ONNV)	Democratic Republic of Congo	*Anopheles* spp.	[63]
	Kenya	*An. funestus*	[64,65]
		An. gambiae	[65]
	Uganda	*An. funestus*	[65,66]
		An. gambiae	[65]
Cache Valley virus (CVV)	Jamaica	*An. grabhami*	[67]
	USA	*An. punctipennis*	[68,69]
		An. quadrimaculatus	[68–71]
		An. walkeri	[69]
Rift Valley fever virus (RVFV)	Kenya	*An. squamosus*	[72]
		Anopheles spp.	[73]
	Madagascar	*An. coustani*	[74]
		An. squamosus	[74]
	Sudan	*An. arabiensis*	[12]
		An. coustani	[12]
Getah virus (GETV)	China	*An. sinensis*	[50,75–78]
	Malaysia	*Anopheles* spp.	[79]
	Rusia	*An. hyrcanus*	[80]
Batai virus (BATV)	Germany	*An. daciae*	[81]
		An. maculipennus s.l.	[82]
		An. messeae	[81]
	Italy	*An. maculipennis*	[83,84]
Sindbis virus (SINV)	Australia	*An. bancroftii*	[85]
		An. meraukensis	[61]
	China	*Anopheles* spp.	[86]
	Germany	*An. maculipennis*	[87]
	Kenya	*An. gambiae*	[88]
Banna virus (BAV)	China	*An. sinensis*	[50,77,89–91]
Zika virus (ZIKV)	Brazil	*An. cruzii*	[92]
	China	*An. sinensis*	[93]
	Senegal	*An. coustani*	[94]
Calovo virus (CVOV)	Austria	*An. maculipennis*	[95,96]
	Croatia	*An. maculipennis* s.l.	[97]
	Slovakia	*An. maculipennis* s.l.	[98]
Jamestown Canyon virus (JCV)	USA	*An. crucians*	[25]
		An. punctipennis	[99,100]
		An. walkeri	[99]
Liao ning virus (LNV)	Australia	*An. amictus*	[59]
		An. annulipes s.l.	[59]
		An. meraukensis	[101]

Bovine fever ephemerovirus (BEFV)	Australia	*An. bancroftii*	[102,103]
	Taiwan	*An. sinensis*	[104]
Saint Louis encephalitis virus (SLEV)	USA	*An. crucians*	[30,105]
		An. quadrimaculatus	[106]
Sagiyama virus (SAGV)	Vietnam	*An. sinensis*	[107]
		An. vagus	[107]
		Anopheles spp.	[107]
Akabane virus (AKAV)	China	*An. sinensis*	[108]
	Taiwan	*An. sinensis*	[104]
	Vietnam	*An. vagus*	[107]
Usutu virus (USUV)	Italy	*An. maculipennis* s.l.	[109–111]
Mapputta virus (MAPV)	Australia	*An. amictus*	[103]
		An. annulipes	[103]
		An. meraukensis	[112]
Ngari virus (NRIV)	Kenya	*An. funestus*	[113]
	Senegal	*An. gambiae* s.l.	[114]
		An. pharoensis	[114]
Bwamba virus (BWAV)	Kenya	*An. coustani*	[115]
	Uganda	*An. funestus*	[66]
Jurona virus	Cambodia	*Anopheles* spp.	[116]
	Senegal	*Anopheles* spp.	[116]
Venezuelan equine encephalitis virus (VEEV)	Mexico	*An. pseudopunctipennis*	[117]
	USA	*An. crucians*	[32]
Nyando virus (NDOV)	Kenya	*An. funestus*	[118]
	Uganda	*An. funestus*	[66]
Kadipiro virus (KDV)	China	*An. sinensis*	[77,119]
Boraceia virus (BORV)	Brazil	*An. cruzii*	[120,121]
Germiston virus (GERV)	Kenya	*An. funestus*	[88]
		An. gambiae	[88]
Chikungunya virus (CHIKV)	Iran	*An. maculipennis* s.l.	[122]
	Senegal	*An. gambiae*	[123]
Bunyamwera virus (BUNV)	Kenya	*An. funestus*	[113]
		An. gambiae	[124]
Orungo virus (ORUV)	Central African Republic	*An. gambiae*	[125]
	Uganda	*An. funestus*	[126]
Tucuruí virus (TUCV)	Brazil	*Anopheles* spp.	[127]
Yada yada virus (YYV)	Australia	*An. annulipes*	[128]
Warrego virus (WARV)	Australia	*An. annulipes* s.l.	[59]
Bluetongue virus (BTV)	Indonesia	*An. vagus*	[129]
Tataguine virus (TATV)	Senegal	*An. gambiae*	[130]
Eubenangee virus (EUBV)	Australia	*An. farauti*	[103]

Near to Cache Valley virus	USA	*An. crucians*	[105]
Aino virus (AINOV)	Taiwan	*Anopheles* spp.	[104]
Barmah Forest virus (BFV)	Australia	*An. amictus*	[60]
Ilheus virus (ILHV)	Brazil	*An. triannulatus*	[131]
Potosi virus (POTV)	USA	*An. punctipennis*	[132]
Bangui virus (BGIV)	Senegal	*An. pharoensis*	[114]
Bozo virus (BOZOV)	Central African Republic	*An. funestus*	[133]
Middelburg virus (MDIV)	Kenya	*An. coustani*	[115]
Epizootic hemorrhagic disease virus (EHDV)	Indonesia	*An. vagus*	[129]
Wallal virus (WALV)	Australia	*An. annulipes* s.l.	[59]
Las Maloyas virus (LMV)	Argentina	*An. albitarsis*	[134]
Wesselsbron virus (WSLV)	Kenya	*An. coustani*	[135]
Arumateua virus (ARTV)	Brazil	*Anopheles* spp.	[127]
Western equine encephalitis virus (WEEV)	USA	*An. punctipennis*	[136]
Iguape virus (IGUV)	Brazil	*An. cruzii*	[131]
Yellow fever virus (YFV)	Panama	*An. neivai*	[137]
Stratford virus (STRV)	Australia	*An. annulipes*	[138]
Tahyna virus (TAHV)	Czech Republic	*An. hyrcanus*	[139]
Peruvian horse sickness virus (PHSV)	Peru	*An. albimanus*	[140]

Table S2. Insect-Specific Viruses (ISVs) detected in wild-caught *Anopheles* mosquitoes worldwide.

Virus name/ abbreviation	Country	*Anopheles* species	References
Anopheles flavivirus (AnFV)	Angola	*Anopheles* spp.	[186]
	Kenya	*An. gambiae*	[135]
		An. gambiae s.l.	[187]
		An. squamosus	[135]
	Turkey	*An. maculipennis* s.l.	[188]
Karumba virus (KRBV)	Australia	*An. meraukensis*	[101,189]
Dianke virus (DKV)	Senegal	*An. funestus*	[190]
		An. gambiae	[190]
		An. pharoensis	[190]
		An. rufipes	[190]
Xinzhou mosquito virus	Cambodia	*Anopheles* spp.	[116]
	China	*An. sinensis*	[191]
	Senega	*Anopheles* spp.	[148]
Culex flavivirus (CxFV)	China	*An. sinensis*	[192]

	Guinea/Mali	*Anopheles* spp.	[193]
Beaumont virus	Australia	*An. annulipes* s.l.	[59]
	Cambodia	*Anopheles* spp.	[116]
	Senegal	*Anopheles* spp.	[116]
Xincheng mosquito virus	Cambodia	*Anopheles* spp.	[116]
	China	*An. sinensis*	[191]
	Senegal	*Anopheles* spp.	[116]
Tanay virus (TANAV)	China	*An. sinensis*	[89,194]
Hubei mosquito virus 2 (HMV2)	China	*An. sinensis*	[49,89]
Wuhan mosquito virus 1	Cambodia	*Anopheles* spp.	[116]
	Senegal	*Anopheles* spp.	[116]
Wuhan mosquito virus 9	Cambodia	*Anopheles* spp.	[116]
	Senegal	*Anopheles* spp.	[116]
Anopheles flavivirus 1 (AnFV1)	Guinea/Mali	*Anopheles* spp.	[193]
	Liberia	*An. gambiae*	[195]
Anopheles flavivirus 2 (AnFV2)	Guinea/Mali	*Anopheles* spp.	[193]
	Liberia	*An. gambiae*	[195]
Culex tritaeniorhynchus rhabdovirus	Cambodia	*Anopheles* spp.	[116]
	Senegal	*Anopheles* spp.	[116]
Anopheles minimus iridovirus (AMIV)	China	*An. minimus*	[50,196]
Anopheles triannulatus ortophasmavirus (AtOPV)	Brazil	*An. lutzi*	[197]
		An. triannulatus	[198]
Australian Anopheles totivirus (AATV)	Australia	*An. annulipes* s.l.	[199]
		An. hinesorum	[199]
Phasi Charoen-like virus	Cambodia	*Anopheles* spp.	[199]
	Senegal	*Anopheles* spp.	[199]
Dairy Swamp virus (DSwV)	Australia	*An. bancroftii*	[189]
Aedes aegypti densovirus (AeDNV)	China	*Anopheles* spp.	[200]
American dog tick phlebovirus	Senegal	*Anopheles* spp.	[199]
Anopheles annulipes orbivirus (AAOV)	Australia	*An. annulipes* s.l.	[199]
Anopheles C virus (AnCV)	Senegal	*An. gambiae* s.l.	[185]
Anopheles cypovirus (AnCPV)	Cambodia	*An. gambiae* s.l.	[185]
Anopheles flavivirus-likesequences1	Senegal	*An. funestus*/*An. gambiae*/*An. rufipes*	[195]
Anopheles flavivirus-likesequences2	Senegal	*An. funestus*/*An. gambiae*/*An. rufipes*	[195]
Anopheles gambiae flavivirus (An(g)FV)	Kenya	*An. gambiae*	[124]

Anopheles hinesorum orbivirus (AHOV)	Australia	*An. hinesorum*	[199]
Anopheles marajoara virus (AnMV)	Brazil	*An. marajoara*	[197]
Anopheles totivirus (AToV)	Liberia	*An. gambiae*	[195]
Anopheles-associated flavivirus (AAFV)	China	*An. sinensis*	[201]
Anopheline-associated C virus (AACV)	France	*An. maculipennis* s.l..	[202]
Beihai mantis shrimp virus 6	China	*An. sinensis*	[89]
Bivens arm virus (BAV)	Cambodia	*Anopheles* spp.	[116]
Bolahun virus variant 1 (BOAV1)	Liberia	*An. gambiae*	[195]
Bolahun virus variant 2 (BOAV2)	Burkina Faso	*An. gambiae*	[195]
Caraipé virus (CRPV)	Brazil	*Anopheles* spp.	[127]
Castlerea virus (CsV)	Australia	*An. annulipes*	[203]
Cerrado partiti-like-virus M1	Brazil	*Anopheles* spp.	[197]
Chapada dielmovirus	Brazil	*Anopheles* spp.	[197]
Coloiado-orthomyxo	Brazil	*Anopheles* spp.	[197]
Coot Bay virus (CBV)	USA	*An. quadrimaculatus*	[204]
Coxipo dielmovirus	Brazil	*Anopheles* spp.	[197]
Culex Bunyavirus 1	China	*An. sinensis*	[205]
Culex pipiens pallens densovirus (CppDNV)	China	*An. sinensis*	[78]
Culex pipiens-associated Tunisia virus (CpATV)	Vietnam	*An. epiroticus*	[206]
Daeseongdong virus 1	Senegal	*Anopheles* spp.	[116]
Eilat virus (EILV)	Israel	*An. coustani*	[207]
Gambie virus (GAMV)	Senegal	*An. funestus/An. gambiae/An. rufipes*	[195]
Golok virus (GOLV)	Indonesia	*An. subpictus*	[129]
Haslams Creek virus (HaCV)	Australia	*An. annulipes* s.l.	[189]
Homalodisca vitripennis reovirus segment S3	Senegal	*Anopheles* spp.	[116]
Ixodes scapularis associated virus 2	Senegal	*Anopheles* spp.	[116]
Jaracatia flavivirus	Brazil	*Anopheles* spp.	[197]
Kampung Karu virus (KPKV)	Malaysia	*An. tessellatus*	[208]
Leanyer virus (LEAV)	Australia	*An. meraukensis*	[209]
Long Pine Key virus (LPKV)	USA	*An. crucians*	[208]
Loreto virus (LORV)	Peru	*An. albimanus*	[210]
Mac Peak virus (McPV)	Australia	*An. farauti*	[189]

Mosquito flavivirus	Kenya	*An. gambiae* s.l.	[187]
Mosquito X virus (MXV)	China	*An. sinensis*	[211]
Mujica picorna-like virus	Brazil	*Anopheles* spp.	[197]
Murrumbidgee virus (MURBV)	Australia	*An. annulipes* s.l.	[59]
Nam Dinh virus (NDiV)	China	*An. sinensis*	[50]
Negev virus (NEGV)	Israel	*An. coustani*	[210]
Nienokoue virus (NiFV)	Cambodia	*Anopheles* spp.	[116]
Oak-Vale virus (OVRV)	Australia	*An. annulipes*	[212]
Omono River virus	Senegal	*Anopheles* spp.	[148]
Orbivirus-like sequences	France	*An. maculipennis* s.l..	[202]
Planococcus citri densovirus	China	*An. sinensis*	[89]
Puerto Almendras virus (PTAMV)	Cambodia	*Anopheles* spp.	[116]
Purunga orbivirus	Brazil	*Anopheles* spp.	[197]
Quang Binh virus (QBV)	China	*An. sinensis*	[75]
Rio Chico virus (RCHV)	Panamá	*An. triannulatus*	[204]
Sunn-hemp mosaic virus	Senegal	*Anopheles* spp.	[116]
Tibet orbivirus (TIBOV)	China	*An. maculatus*	[213]
Wuhan fly virus 1	China	*An. sinensis*	[89]
Wuhan mosquito virus 6	China	*An. sinensis*	[205]
Wuhan spider virus	Senegal	*Anopheles* spp.	[116]
Wutai mosquito phasivirus	China	*An. sinensis*	[205]
Yunnan Culex flavivirus (YNCxFV)	China	*An. sinensis*	[214]
Yunnan Culex-related flavivirus	China	*An. sinensis*	[50]
Yunnan orbivirus (YUOV)	China	*An. sinensis*	[50]

Table S3. Summary of laboratory studies that evaluated virus infection and transmission in *Anopheles* mosquitoes.

Virus name/ abbreviation	Category	*Anopheles* species	Stage	Infection Route/ virus dose	Results of laboratory studies	References
Aedes aegypti densovirus (AeDNV)	ISV	*An. gambiae*	Larvae	OT/ 500 µl of a 1: 4 dilution of virus stock of transducing particles	Infection 17%, but not disseminate further anal papillae, TR not determined	[156]
Anopheles C virus (AnCV)	ISV	*An. coluzzii*	Adult	OT, virus dose not available	Transovarial intraembryonic transmission, infection, TR not determined	[157]
Anopheles cypovirus (AnCPV)	ISV	*An. coluzzii*	Adult	OT, virus dose not available	Transovarial intraembryonic transmission, infection, TR not determined	[158]
Anopheles gambiae densovirus (AgDNV)	ISV	*An. gambiae*	Adult	IT, 10^{7} viral genome equivalents per ml (vge/mL)	Paratransgenesis, infection at 7 dpi, venereal transmission in males, IR and TR not determined	[159]
	ISV	*An. gambiae*	Larvae	OT, virus dose not available	Paratransgenesis, infection, dissemination and vertical transmission, IR and TR not determined	[160]
Bunyamwera virus (BUNV)	Probable arbovirus	*An. gambiae*	Adult	BM, 10^{10} PFU/mL	IR 38%, transmission 71% at 14 dpi (mice)	[144]
Buttonwillow virus (BUTV)	Arbovirus	*An. freeborni*	Adult	OT, 107.9 PFU/mL	Infection 4/10, but no transmission (rabbit)	[161]
Bwamba virus (BWAV)	Probable arbovirus	*An. gambiae*	Adult	BM, $10^{6.7}$ PFU/mL	Infection at 6 and 14 dpi (rates not available), TR not determined	[145]
Cache Valley virus (CVV)	Arbovirus	*An. quadrimaculatus*	Adult	BM, $10^{5.2-6.2}$ PFU/mL	IR 100%, transmission 20% at 7 dpi; IR 100%, transmission 33% at 14 dpi (mice)	[142]
		An. quadrimaculatus	Adult	BM, $ICLD_{50}$ ($10^{4,8}$)	IR 100%, TR 0% at 10-19 dpi	[143]
		An. punctipennis	Adult	BM, $ICLD_{50}$ ($10^{5.3}$)	IR 85%, TR 30% at 14-18 dpi	[143]

Chikungunya virus (CHIKV)	Arbovirus	*An. albimanus*	Adult	BM, $10^{8.0.}$ $TCID_{50}$/mL	Infection 7/7 at 13 dpi, infection until 3 weeks dpi, transmission 0% (mice)	[162]
		An. stephensi	Adult	BM, ICLD50 106.7 PFU/mL (post-infection)	IR 50% at 8 dpi, 54% at 10 dpi, effective transmission to mice (1/8)	[163]
Dianke virus (DKV)	ISV	*An. gambiae*	Adult	BM, 1.6×10^{7} copies/mL	Capable of disseminate virus at 20 dpi, but no transmission (saliva)	[164]
Eastern equine encephalitis virus (EEEV)	Arbovirus	*An. albimanus*	Adult	BM, (10^{7}-10^{8}) $ICLD_{50}$	Infection rate not determined, transmission 100% at 7 dpi, 90% at 11 dpi (mice)	[152]
		An. quadrimaculatus	Adult	BM, (10^{7}-10^{8}) $ICLD_{50}$	Infection rate not determined, transmission 40% at 10 dpi, 50% at 11 dpi (mice)	[152]
		An. quadrimaculatus	Adult	BM, 10^{6}PFU/mL	IR 54%, TR 13% at 7 dpi; IR 24%, TR 0% at 14 dpi (saliva)	[165]
		An. punctipennis	Adult	BM, 10^{6}PFU/mL	IR 100% at 7 dpi and at 14 dpi, TR 0% at 7 dpi and at 14 dpi (saliva)	[165]
Eilat virus (EILV)	ISV	*An. gambiae*	Adult	IT, 10^{7}PFU/mL	Infection only in posterior midgut at 7dpi, not disseminated, IR and TR not determined	[166]
Flock house virus (FHV)	ISV	*An. gambiae*	Adult	IT, $5X10^{4}$–$5X10^{5}$ PFU/mosquito	Infection at 10 dpi, IR and TR not determined	[167]
		An. gambiae	Adult	IT, $10^{5.3}$ $10^{3.5}$ PFU/mosquito	Infection at 7 dpi and 10 dpi, IR and TR not determined	[168]
		An. stephensi	Adult	IT, $10^{5.3}$ $10^{3.5}$ PFU/mosquito	Infection at 10 dpi, IR and TR not determined	[168]
Friend murine leukemia virus (FLV)	Other	*An. stephensi*	Adult	BM, virus dose not available	Infection at 78 hrs dpi, TR not determined	[169]
Hepatitis B virus (HBV)	Other	*An. stephensi*	Adult	BM, 1,500 pg/mL	IR 40% at 48 hrs dpi, but at 72 hrs dpi was negative, TR 0% (saliva)	[170]

Mayaro virus (MAYV)	Arbovirus	*An. freeborni*	Adult	BM, $10^{6.8}$ FFU/mL	IR 37%, TR 67% at 7 dpi; IR 0%, TR 0% at 14 dpi (saliva)	[13]
		An. quadrimaculatus	Adult	BM, 10^{7} FFU/mL	IR 79%, TR 50% at 7 dpi; IR 100%, TR 0% at 14 dpi (saliva)	[13]
		An. stephensi	Adult	BM, 10^{7} FFU/mL	IR 71%, TR 12,5% at 7 dpi; IR 79%, TR 0% at 14 dpi (saliva)	[13]
		An. gambiae	Adult	BM, $10^{7.1}$ FFU/mL	IR 75%, TR 0% at 7 dpi; IR 100%, TR 100% at 14 dpi (saliva)	[13]
		An. quadrimaculatus	Adult	BM, $10^{7.87}$-$10^{8.39}$ PFU/mL	IR 82%, TR 25% at 7 dpi; IR 74%, TR 48% at 14 dpi (saliva)	[171]
Myxoma virus (MYXV)	Other	*An. atroparvus*	Adult	BM rabbit, virus dose not available	Vector can retain its infectivity for periods up to 220 dpi, and it can infect rabbits	[154]
Ngari virus (NRIV)	Arbovirus	*An. gambiae*	Adult	BM, 1010 PFU/mL	IR 38% 14 dpi, transmission 100% (mice)	[144]
Northway virus (NORV)	Probable arbovirus	*An. freeborni*	Adult	IT, $10^{3.2}$ PFU/mosquito	IR 35% (dpi not available), TR 14% at 7-23 dpi (saliva)	[172]
O'nyong'nyong virus (ONNV)	Arbovirus	*An. gambiae*	Adult	BM, 10 5.0-6.9 PFU/mL	IR 75% at 7 dpi with recombinant virus, TR not determined	[4]
		An. gambiae	Adult	BM, $10^{5.5}$ PFU/mL	Infection, IR not available, TR not determined	[146]
		An. gambiae	Adult	IT, 10^{1}-$10^{3.5}$ PFU/mL; BM 10^{2}-10^{5} PFU/mL	Limited infection and spread, with no differences between transgenic and wild mosquitoes, TR 0%	[147]
		An. gambiae	Adult	BM, $10^{5.0}$-$10^{5.5}$. PFU/mL	IR 78%, DR 15% at 6 dpi; IR 84%, DR 25% at 8 dpi of recombinant virus, TR not determined	[148]
		An. gambiae	Adult	BM, $10^{5.0}$-$10^{5.5.}$ $TCID_{50}$/mL	IR 75%, TR 0% at 7 dpi; IR 95%, TR 57% at 14 dpi (saliva)	[149]

Rift Valley fever virus (RVFV)	Arbovirus	*An. bradley-crucians*	Adult	IT, $10^{1.6}$ PFU/mL	IR 83%, transmission 0% at 14 dpi (Hamster)	[173]
		An. stephensi	Larvae	IT, 10^{2} PFU/mL	IR not available, transmission 83% at 7-10 dpi (hamster), when the virus is inoculated as an adult, none of the mosquitoes transmit it	[174]
		An. coustani	Adult	BM, 2.25 × 10^{8} PFU/mL	IR 50%, TR 100% at 8 dpi (saliva)	[150]
		An. stephensi	Adult	IT, 10^{2} PFU/mL	IR 46%, transmission RVFV (hamster) (co-infection RVFV and sporozoite of *Plasmodium berghei*)	[175]
Saint Louis encephalitis virus (SLEV)	Arbovirus	*An. quadrimaculatus*	Adult	BM, virus dose not available	Infection (IR not determined), transmission 0% (mice, monkey)	[151]
		An. maculipennis freeborni	Adult	BM, virus dose not available	Infection (IR not determined), transmission 0% at 8 dpi	[176]
Semliki Forest virus (SFV)	Arbovirus	*An. albimanus*	Adult	BM, initial virus dose not available	IR not determined, transmission 24% at 14 dpi (mice)	[177]
		An. albimanus	Adult	BM, $10^{5.9}$ ICLD $_{50}$/mL	IR 92% at 10 dpi; transmission to mice	[178]
		An. quadrimaculatus	Adult	BM, $10^{6.7}$ ICLD $_{50}$/mL	IR 100% at 10 dpi; transmission to mice	[178]
		An. freeborni	Adult	BM, $10^{3.0.-4.1}$ ICLD $_{50}$/mL	Transmission at 12 dpi (4/12 mice)	[179]
		An. stephensi	Adult	BM, $10^{4.3-5.0}$ ICLD $_{50}$/mL	Transmission at 13 dpi (10/10 mice)	[179]
		An. atroparvus	Adult	BM, $10^{5.7-6.2}$ ICLD $_{50}$/mL	Transmission at 10 dpi (4/20 mice)	[179]
		An. sundaicus	Adult	BM, $10^{5.2-5.6}$ ICLD $_{50}$/mL	Transmission at 10 dpi (3/15 mice)	[179]
Sindbis virus (SINV)	Arbovirus	*An. albimanus*	Adult	BM, virus dose not available	Transmission at 14 dpi (10/10 mice)	[180]
		An. freeborni	Adult	IT, 100-1000 PFU	Infection, but low virus levels, IR and TR not determined	[181]
Sunguru virus (SUNV)	Probable arbovirus	*An. gambiae*	Adult	BM, $10^{5.5}$ PFU/mL	IR 47%, TR 0% (saliva)	[182]

Tensaw virus (TENV)	Arbovirus	*An. quadrimaculatus*	Adult	BM, $10^{4.1}$ ICLD $_{50}$/mL	IR 100% at 10 and 20 dpi, transmission at 14 dpi (3/15 mice)	[141]
		An. albimanus	Adult	BM, $10^{3.9}$ ICLD $_{50}$/mL	IR 94% at 12 dpi, transmission at 12 dpi (4/13 mice)	[141]
		An. maculatus	Adult	BM, $10^{2.9-4.0}$ ICLD $_{50}$/mL	IR 100% at 6 dpi, transmission 0% (mice)	[141]
Thai-strain densovirus (AThDNV)	ISV	*An. minimus*	Larvae	OT, virus dose not available	Infection and vertical transmission, IR and TR not determined	[183]
Venezuelan equine encephalitis virus (VEEV)	Arbovirus	*An. albimanus*	Adult	BM, $10^{7.2}$ LD $_{50}$/mL	Infection until 13 dpi, transmission at 14 dpi (1/6 mice)	[184]

Abbreviations: ISV, Insect-Specific Virus; **BM**, mosquitoes were infected by infectious blood-meal; **IT**, mosquitoes were infected by intrathoracic inoculation; **OT**, mosquito were infected by other via; **PFU**, Plaque forming units; **FFU**, Focus forming units; **vge**, viral genome equivalents; **$TCID_{50}$**, tissue culture infectious dose 50%; **ICLD50**, intracerebral lethal dose 50%; **LD**, lethal dose, 50%; **IR**, Infection rate is the percentage of engorged females with viral particles in the body; **DR**, Dissemination rate is the percentage of engorged females with viral particles in legs/wings; **TR**, Transmission rate is calculated as percentage of engorged females with viral particles in the saliva/ salivary glands; **dpi**, days post-infection; **hrs**, hours.

*The results presented in this table reflect those reported in the studies with no modifications.
